# Four New Species of Larval *Charletonia* and *Leptus* (Acari: Trombidiformes: Erythraeidae), with a Checklist of the Two Genera and Their Hosts from China †

**DOI:** 10.3390/insects13121154

**Published:** 2022-12-14

**Authors:** Si-Yuan Xu, Tian-Ci Yi, Jian-Jun Guo, Dao-Chao Jin

**Affiliations:** 1 Institute of Entomology, Guizhou University, Guiyang 550025, China; syxuxsy@163.com (S.-Y.X.); tcyi@gzu.edu.cn (T.-C.Y.); jjguo@gzu.edu.cn (J.-J.G.); 2 The Guizhou Provincial Key Laboratory for Plant Pest Management of Mountainous Region, Guiyang 550025, China; 3 The Scientific Observing and Experimental Station of Crop Pest in Guiyang, Ministry of Agriculture P. R. China, Guiyang 550025, China

**Keywords:** host, biodiversity hotspot, ectoparasite, Callidosomatinae, Leptinae

## Abstract

**Simple Summary:**

The bright red color of Erythraeid mites is conspicuous. The Erythraeid larvae are usually ectoparasitic on arthropods and easily observed. Both the genera *Charletonia* and *Leptus* are distributed worldwide. *Charletonia* has 86 species and *Leptus* has more than 240 species based on larvae, respectively. To date, two species of the genus *Charletonia* and 11 species of the genus *Leptus* have been reported from China. Here, four new species, *Charletonia rectangia* Xu and Jin **sp. nov.** collected from tropical rainforests in the Guangxi Province and Yunnan Province, *Leptus* (*Leptus*) *bomiensis* Xu and Jin **sp. nov.** from the Tibet Autonomous Region, where the altitude ranges from 2673 to 3374 m, *Leptus* (*Leptus*) *longisolenidionus* Xu and Jin **sp. nov.** from jungles in the Hainan Province (Hainan Island), and *Leptus* (*Leptus*) *striatus* Xu and Jin **sp. nov.** from Xishuangbanna tropical rainforests in the Yunnan Province. We believe that this study will contribute to further research on the taxonomy and phylogeny of the family.

**Abstract:**

Four new species, *Charletonia rectangia* Xu and Jin **sp. nov.**, *Leptus* (*Leptus*) *bomiensis* Xu and Jin **sp. nov.**, *Leptus* (*Leptus*) *longisolenidionus* Xu and Jin **sp. nov.**, and *Leptus* (*Leptus*) *striatus* Xu and Jin **sp. nov.** are described and illustrated based on larvae. All four new species are from biodiversity hotspots, *L*. (*L*.) *bomiensis* **sp. nov.** from the Eastern Himalayas biodiversity hotspot, while the other three species from the Indo–Burma biodiversity hotspot.

## 1. Introduction

The genus *Charletonia* Oudemans, 1910 is globally distributed and includes 86 species based on larvae [1]. The hosts of *Charletonia* larvae were recorded in three animal classes (Arachnida, Insecta, and Mammalia), 16 orders, and 45 families [2,3,4,5,6,7,8,9,10,11,12]. The most common hosts are arthropods, especially those of Araneae (Arachnida), Coleoptera (Insecta), Hemiptera (Insecta), Lepidoptera (Insecta), and Orthoptera (Insecta).

Nowadays, only two species of *Charletonia* are reported in China: *C*. *banksi* Southcott, 1966 (synonym *C*. *hunanensis* Zheng, 1996) based on the larval stage [1,13] and *C*. *taiwanensis* Tsai and Chow, 1988 based on the larvae and active post-larval instars [14]. The hosts of *C. banksi* are the dragonflies (Libellulidae) and damselflies (Megapodagriidae) in China [13], and in Australia, are orthopteran insects in Acrididae, *Hepalieus gracilis*, *Goniaea vocans*, *Nomaducris guttulosa* and *Oedaleus australis* [6,11]. The host of *C*. *taiwanensis* is also a species of Acrididae, *Chondracris rosea* [14].

The genus *Leptus* Latreille, 1796 was reported on all continents except for Antarctica [15], with more than 240 described species from the larval stage [15,16,17,18,19,20,21,22]. The hosts of this genus were reported from 101 families belonging to six classes (Insecta, Arachnida, Diplopoda, Entognatha, Aves, and Mammalia) and 28 Orders [2,3,4,18,23,24,25,26,27,28,29,30,31,32,33,34,35]. However, the common hosts of its larvae are arthropods, especially Araneae, Coleoptera, Diptera, Hemiptera, Lepidoptera, Opiliones, and Orthoptera [2,16].

To date, a total of 11 species of the genus *Leptus* have been reported based on larvae from China, of which only three species have host records and all the hosts are insects [13,36,37,38,39,40].

In this paper, *Charletonia rectangia* Xu and Jin **sp. nov.**, *Leptus (Leptus) bomiensis* Xu and Jin **sp. nov.**, *Leptus (Leptus) longisolenidionus* Xu and Jin **sp. nov.**, and *Leptus (Leptus) striatus* Xu and Jin **sp. nov.** are described and illustrated based on larvae from China. All the specimens of the four new species were collected from biological hotspots [41,42,43].

## 2. Materials and Methods

Moths were collected by a light trip, harvestmen were captured on leaves or branches late at night, and other insects were collected using an insect net in the field and subsequently preserved in small vials containing 75% ethanol. Erythraeid larval specimens on insects or harvestmen were detached by a fine brush under a stereomicroscope. Then, the larval specimens were prepared with Oudemans’ fluid and mounted in Hoyer’s medium. Figures were drawn with the aid of a drawing tube attached to a Nikon Eclipse Ni-E microscope. The distribution map was prepared with Arcmap 10.3. The terminology and abbreviations are adapted from Bassini-Silva et al. [18,44], Jacinavicius et al. [45], Haitlinger and Saboori [46], Šundić et al. [47] and Xu et al. [48]. Measurements are expressed in micrometers (μm). The SD, standard deviation, keeps two decimal fractions.

## 3. Results

### 3.1. New Species

*Charletonia* Oudemans, 1910

**Figure 1:**
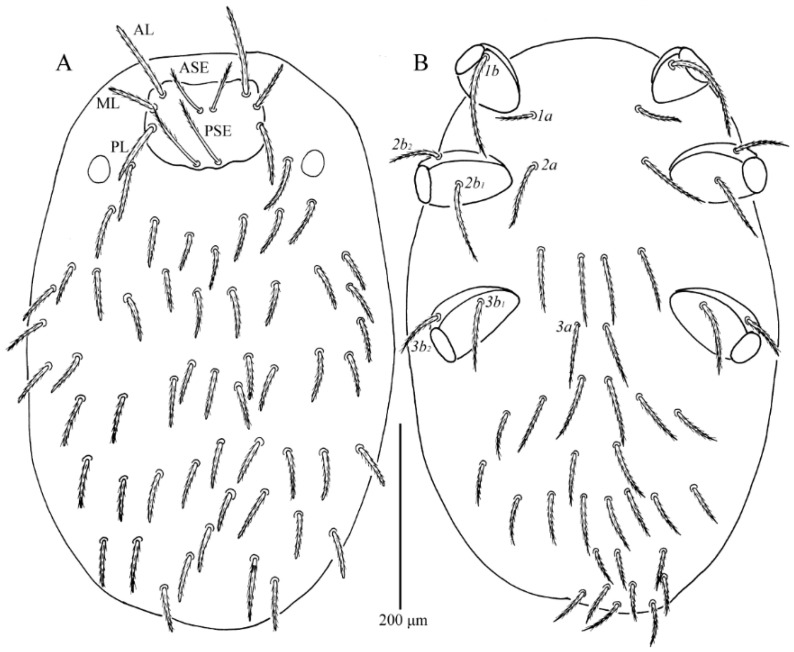
*Charletonia rectangia* **sp. nov.**, larva. (**A**). Dorsal view of idiosoma. (**B**). Ventral view of idiosoma.

**Figure 2:**
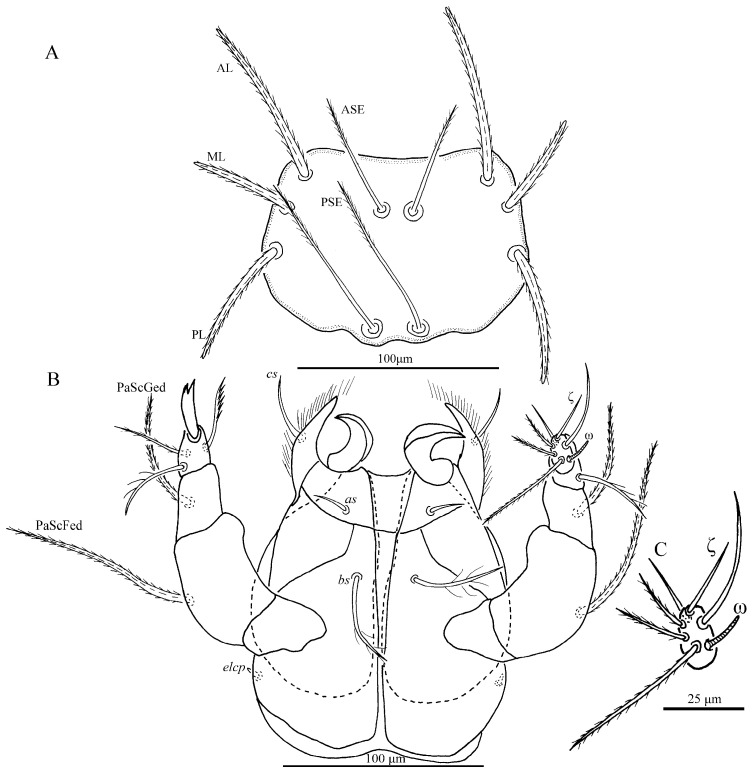
*Charletonia rectangia* **sp. nov.**, larva. (**A**). Scutum. (**B**). Ventral view of gnathosoma. (**C**). Ventral view of palptarsus.

**Figure 3:**
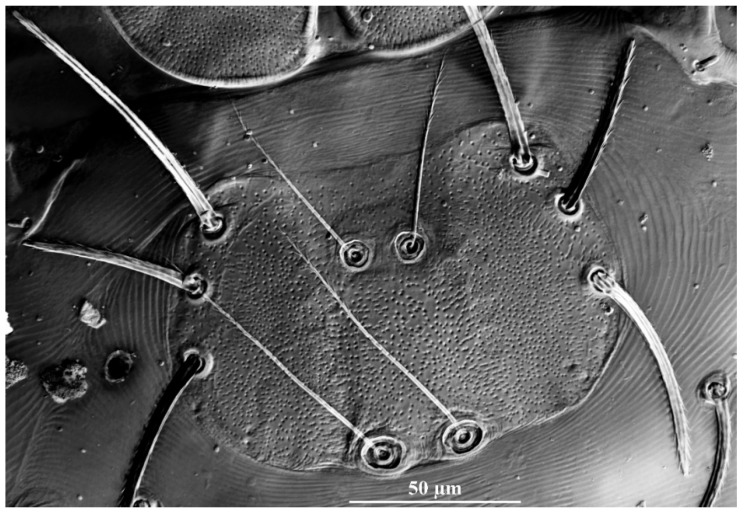
*Charletonia rectangia* **sp. nov.**, larva. Showing ASE and PSE shape and outline of scutum.

**Figure 4:**
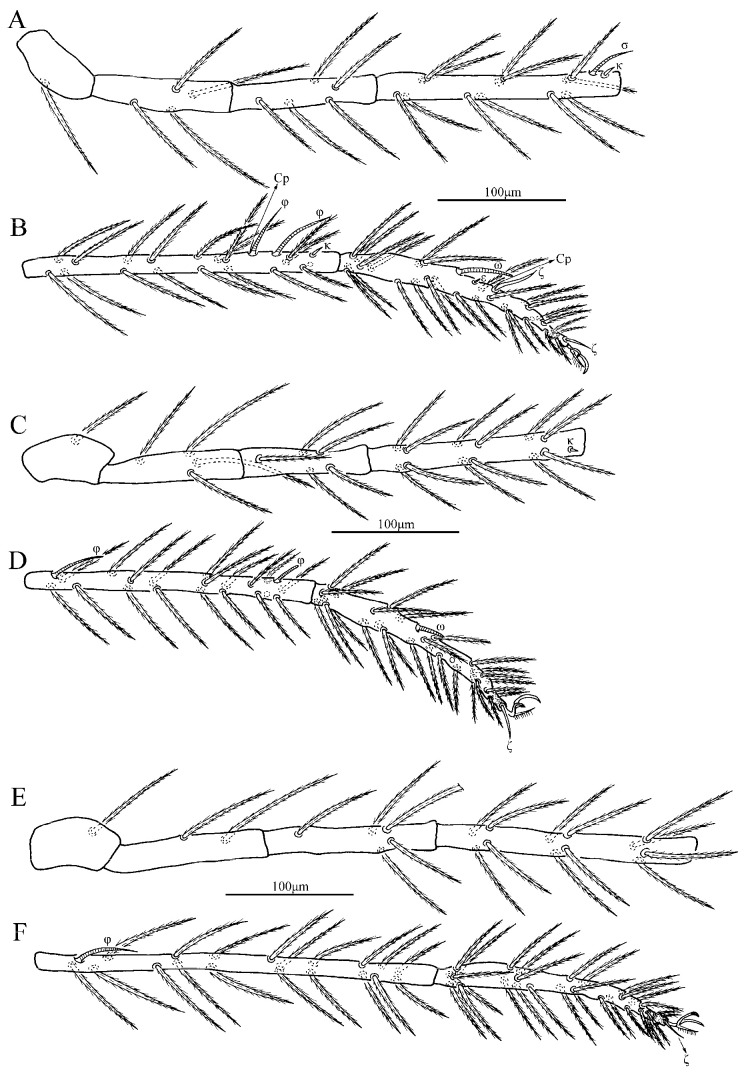
*Charletonia rectangia* **sp. nov.**, larva. (**A**). Leg I, trochanter—genu. (**B**). Leg I, tibia—tarsus. (**C**). Leg II, trochanter—genu. (**D**). Leg II, tibia—tarsus. (**E**). Leg III, trochanter—genu. (**F**). Leg III, tibia—tarsus.

**Figure 5:**
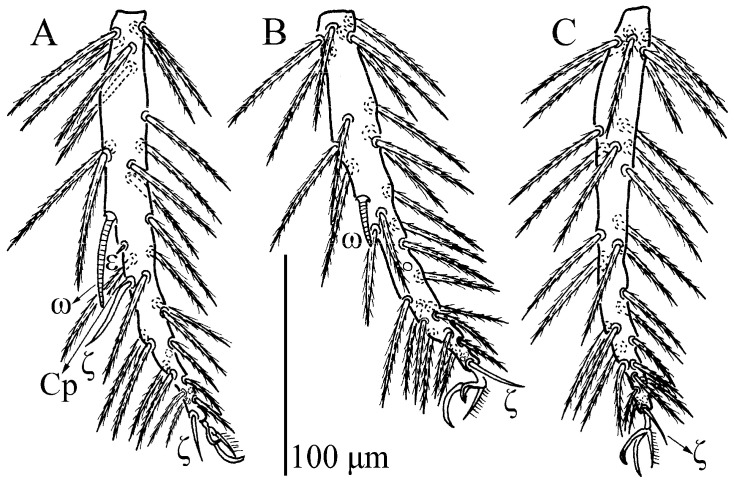
*Charletonia rectangia* **sp. nov.**, larva. (**A**). Leg I, tarsus. (**B**). Leg II, tarsus. (**C**). Leg III, tarsus.

*Charletonia rectangia* Xu and Jin **sp. nov.** (Figure 1, Figure 2, Figure 3, Figure 4 and Figure 5)

**Diagnosis (larva).** Four setae between coxae II and III; gnathosoma with two hypostomalae; solenidion on Ge I distal to most distal normal setae; ASE posterior to ML bases and very close to ML bases, ASE and PSE with fine barbs on distal halves; Ti I 241–265; Ti III 305–331.

**Description.** Dorsum. Idiosoma with 54 (fD = 54–58 in paratypes) barbed setae, a pair of setae located between scutum and eyes at level with PSE bases (Figure 1A). Scutum about rectangular outline with rounded angles, anterior margin slightly concave, lateral margins almost straight, posterior margin wavy near the base of PSE (Figure 1A, Figure 2A and Figure 3). Scutum with three pairs of normal setae (AL, ML and PL), and two pairs of sensilla (ASE and PSE), AL, ML and PL are completely barbed, ASE and PSE with barbs in distal half. PSE is much longer than ASE, AL > PL > ML (Table 1). ASE bases are posterior but very close to the level of ML bases, PSE near the posterior margin of scutum.

Venter (Figure 1B). All ventral setae, including coxal fields, are barbed and acute (Figure 1B). Five pairs of intercoxal setae (paired intercoxal setae *1a*, *2a* and *3a*, and two pairs of unnamed setae between II and III), *1a* posterior to level of the posterior edge of coxae I, *2a* at a line with anterior edges of coxae II, *3a* between coxae III; 25 setae behind coxae III (fV = 24–26 in paratypes). Intercoxal setae II (*2a*) is slightly longer than intercoxal setae III (*3a*), *2a* and *3a* both longer than *1a*. Five pairs of coxalae (*1b*, *2b_1_*, *2b_2_*, *3b_1_* and *3b_2_*), *1b* longest, *2b_1_* longer than *3b_1_*, *2b_2_* and *3b_2_* subequal, *2b_1_* longer than *2b_2_*, *3b_1_* longer than *3b_2_* (Table 1).

Gnathosoma (Figure 2B). With two nude galealae (*cs*), two nude anterior hypostomalae (*as*) and two subcapitular setae (*bs*) with few setules; *bs* longer than *cs* and much longer than *as* (Table 1). Hypostomal lip with fimbriated. Palpfemur and palpgenu each with one barbed, pointed dorsal seta (PaScFed and PaScGed). Palptibia with one seta on ventral surface, this seta with few setules; palptibia with one barbed dorsal seta, and one brush-like dorsal seta; odontus bifid. Palptarsus with seven setae, of which, three barbed, two nude, one solenidion (ω) and one eupathidium (ζ). fPp = 0-B-B-3B_2_-3B2Nωζ. Palpal supracoxal seta (*elcp*) peg-like.

Legs (Figure 4 and Figure 5). With seven segments (femora divided). IP = 2901–3081 (Holotyp and seven paratypes). Dorsum of coxa I with a supracoxal seta (*eI*) which is peg-like with a rounded tip. Anterior claw hook-like, posterior claw pulvilliform with few ciliations, and empodium claw-like or falciform. Normal setae on legs barbed and pointed. Leg setal formula: leg I: Cx—1n; Tr—1n; Bfe—4n; Tfe—5n; Ge—1σ, 1κ, 12n; Ti—2φ, 1κ, 1Cp, 18n; Ta—1ω, 1ε, 2ζ, 29n (27n in the paratype numbered g, both sides). leg II: Cx—2n; Tr—1n; Bfe—4n; Tfe—5n; Ge—1κ, 12n; Ti—2φ, 19n; Ta—1ω, 1ζ, 30n. leg III: Cx—2n; Tr—1n; Bfe—2n; Tfe—5n; Ge—12n; Ti—1φ, 19n; Ta—1ζ, 30n. The lengths of the legs were measured from coxae to tarsus (Table 1).

**Etymology.** The new species is named after the rectangular-like shape of the scutum.

**Types.** Holotype, larva, an unknown long-horned grasshopper of Tettigoniidae (Orthoptera), collected by Si-yuan Xu on 10 May 2016, from Xishuangbanna National Natural Reserve (Altitude: 672 m), Yunnan Province, China. Paratypes: two larvae, the same data as the holotype; one larva, an unidentified mantis (Mantodea), collected by Si-yuan Xu on 7 May 2016, from Xishuangbanna National Natural Reserve (Altitude: 627 m); one larva, an unknown grasshopper of Acrididae (Orthoptera), collected by Si-yuan Xu on 7 May 2016, from Xishuangbanna National Natural Reserve (Altitude: 627 m); one larva, an unidentified moth (Lepidoptera), collected by Si-yuan Xu on 24 April 2018, from Xishuangbanna National Natural Reserve (Altitude: 633 m); one larva, an unidentified stick (Phasmatodea), collected by Si-yuan Xu on 28 April 2018, from Xishuangbanna National Natural Reserve (Altitude: 732 m); one larva, an unknown beetle of Chrysomelidae (Coleoptera), collected by Yan Jiang on 18 April 2019, from Nonggang National Natural Reserve (Altitude: 293 m), Guangxi Province, China.

The holotype and paratypes are deposited in the Institute of Entomology, Guizhou University, Guiyang, China (GUGC).

**Distribution.** China: Guangxi and Yunnan Province.

**Remarks.** According to the keys by Hakimitabar and Saboori [1], *C. rectangia* **sp. nov.** falls into the *brunni* species group (Four setae between coxae II and III), and *buforania* species subgroup (*Sigma* (σ) on Ge I placed in the distal half of the segment after the most distal normal seta, ASE posterior to or the same level with ML). This subgroup includes nine species, of which, *C*. *alvedae* Haitlinger, 2000 was from Peru [49]; *C*. *buforania* (Womersley, 1934) [5] and *C*. *striaticeps* Southcott, 1991 [6] from Australia; *C*. *lankensis* Southcott, 1988 from India and Sri Lanka [50,51]; *C*. *rajmundi* Haitlinger, 2007 from South Africa [52]; *C*. *stekolnikovi* Hakimitabar and Saboori, 2011 [53] and *C*. *terianae* Hakimitabar, Saboori and Seiedy, 2013 [54] from Iran; *C*. *villingensis* Haitlinger, 2007 from Maldives [51]; *C*. *womersleyi* Southcott, 1966 from Belgium and Great Britain [5].

*Charletonia rectangia* **sp. nov.** differs from *C*. *alvedae* by the longer Ti I (241–265 vs. 192), Ti II (219–231 vs. 178), Ti III (305–331 vs. 220), Ge I (175–199 vs. 146), Ge II (152–168 vs. 140), Ge III (183–203 vs. 140), *1b* (115–119 vs. 76).

*Charletonia rectangia* **sp. nov.** differs from *C*. *buforania* by the longer Ti I (241–265 vs. 155), Ti III (305–331 vs. 192), Ge I (175–199 vs. 125), Ge III (183–203 vs. 121), W (121–141 vs. 86–95), AL (80–96 vs. 34–42), PL (64–79 vs. 32–42).

*Charletonia rectangia* **sp. nov.** differs from *C*. *lankensis* by the longer Ti I (241–265 vs. 98–110), Ti II (219–231 vs. 82–92), Ti III (305–331 vs. 122–132), Ge I (175–199 vs. 84–92), Ge II (152–168 vs. 68–80), Ge III (183–203 vs. 80–92), W (121–141 vs. 92–108), AL (80–96 vs. 40–58), PL (64–79 vs. 42–52), ASE (60–71 vs. 40–49), *1b* (115–119 vs. 50–64).

*Charletonia rectangia* **sp. nov.** differs from *C*. *rajmundi* as follows: posterior half of scutum in *C*. *rectangia* **sp. nov.** polygonal with wavy edges, while semicircular in *C*. *rajmundi*; shape of ASE and PSE (barbed about distal half vs. nude), the number of setae in fD formula (54–58 vs. 148–152); the shorter Ti I (241–265 vs. 284–304) and Ti III (305–331 vs. 346–364), IP (2901–3081 vs. 3158–3444).

*Charletonia rectangia* **sp. nov.** differs from *C*. *stekolnikovi* by the longer Ti I (241–265 vs. 134–136), Ti II (219–231 vs. 129), Ti III (305–331 vs. 176), W (121–141 vs. 90–91), AL (80–96 vs. 48–50), PL (64–79 vs. 54–55), and *1b* (115–119 vs. 77–79).

*Charletonia rectangia* **sp. nov.** differs from *C*. *striaticeps* as follows: the dorsal of cheliceral bases with striations (no vs. yes), scutum with a prominent reticular pattern (no vs. yes); the longer Ti I (241–265 vs. 120–169), Ti II (219–231 vs. 111–144), and Ti III (305–331 vs. 162–222).

*Charletonia rectangia* **sp. nov.** differs from *C*. *terianae* by the number of setae in fD formula (54–58 vs. 78), the longer Ti I (241–265 vs. 101–119), Ti II (219–231 vs. 94–109), Ti III (305–331 vs. 129–153), L (95–109 vs. 69–87), W (121–141 vs. 79–94), AL (80–96 vs. 45–54), and *1b* (115–119 vs. 67–87).

*Charletonia rectangia* **sp. nov.** differs from *C*. *villingensis* by the longer Ti I (241–265 vs. 116), Ti II (219–231 vs. 100), Ti III (305–331 vs. 144), IP (2901–3081 vs. 1694), W (121–141 vs. 98), AL (80–96 vs. 52), ASE (60–71 vs. 32), and *1b* (115–119 vs. 72).

*Charletonia rectangia* **sp. nov.** differs from *C*. *womersleyi* by the longer Ti I (241–265 vs. 110), Ti III (305–331 vs. 140), Ta I (181–198 vs. 98), Ta III (180–196 vs. 104), W (121–141 vs. 107), AW (84–91 vs. 66), PW (115–122 vs. 92).

*Leptus* Latreille, 1796

**Figure 6:**
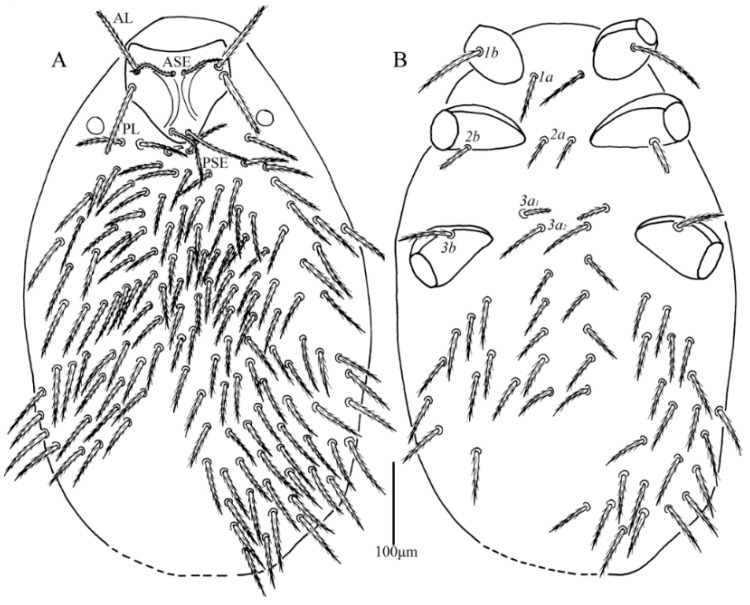
*Leptus (Leptus) bomiensis* **sp. nov.**, larva. (**A**). Dorsal view of idiosoma. (**B**). Ventral view of idiosoma.

**Figure 7:**
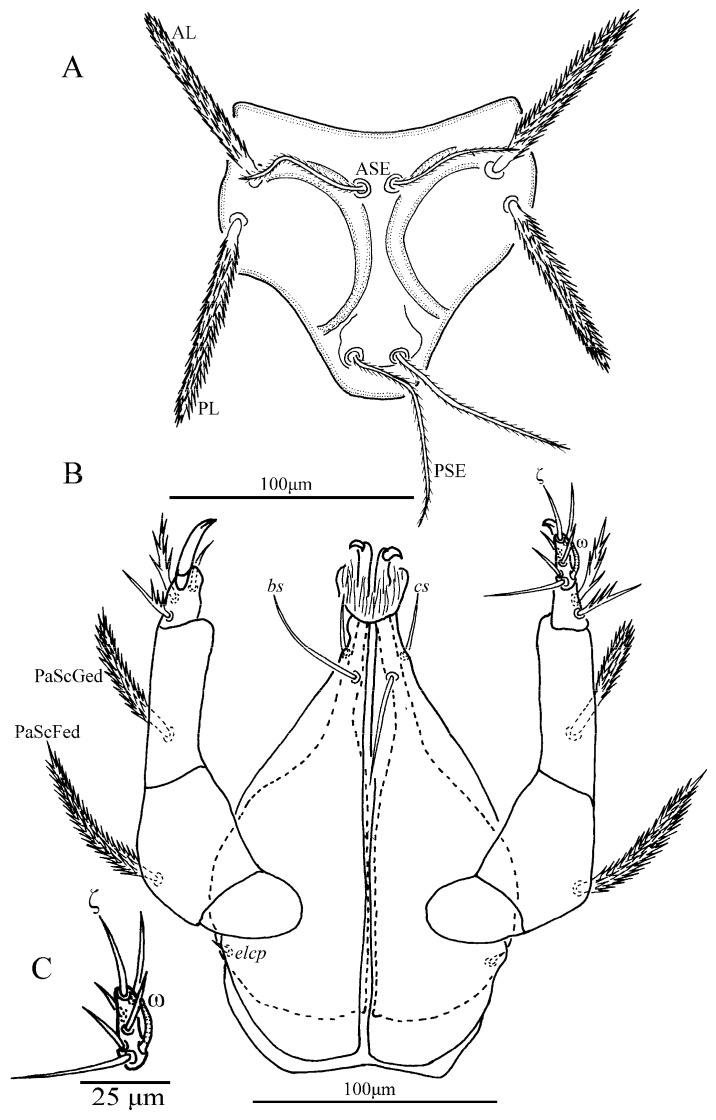
*Leptus (Leptus) bomiensis* **sp. nov.**, larva. (**A**). Scutum. (**B**). Ventral view of gnathosoma. (**C**). Ventral view of palptarsus.

**Figure 8:**
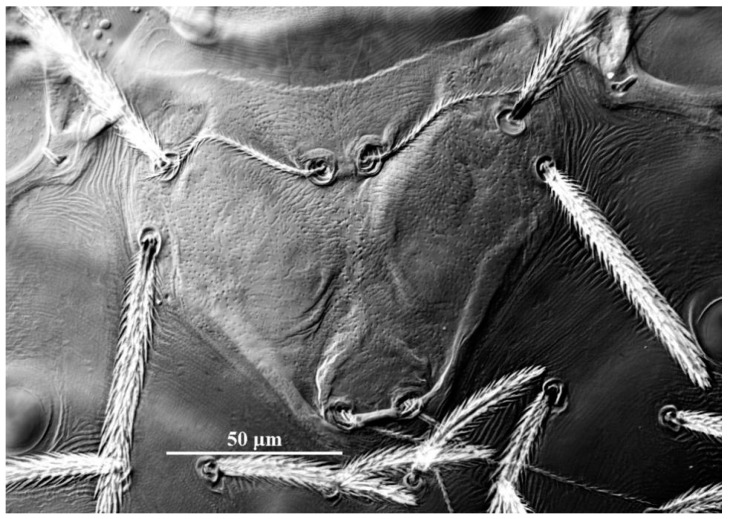
*Leptus (Leptus) bomiensis* **sp. nov.**, larva. Showing ASE and PSE shape and outline of scutum.

**Figure 9:**
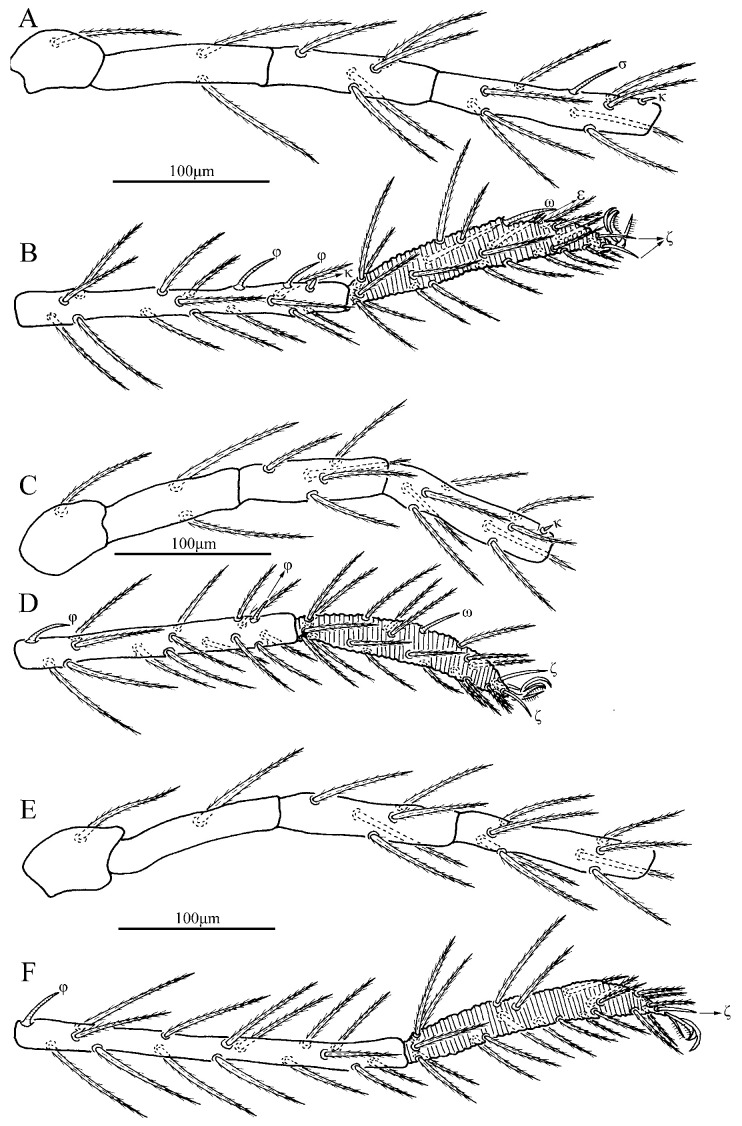
*Leptus (Leptus) bomiensis* **sp. nov.**, larva. (**A**). Leg I, trochanter—genu. (**B**). Leg I, tibia—tarsus. (**C**). Leg II, trochanter—genu. (**D**). Leg II, tibia—tarsus. (**E**). Leg III, trochanter—genu. (**F**). Leg III, tibia—tarsus.

**Figure 10:**
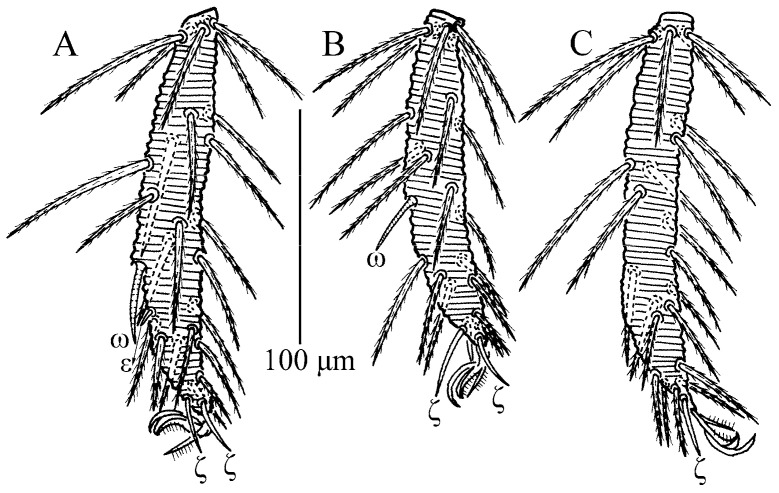
*Leptus (Leptus) bomiensis* **sp. nov.**, larva. (**A**). Leg I, tarsus. (**B**). Leg II, tarsus. (**C**). Leg III, tarsus.

*Leptus (Leptus) bomiensis* Xu and Jin **sp. nov.** (Figure 6, Figure 7, Figure 8, Figure 9 and Figure 10)

**Diagnosis (larva).** ASE and PSE with fine barbs throughout the length; gnathosoma with two hypostomalae; palpfemur and palpgenu each with one barbed seta on the dorsal surface (PaScFed and PaScGed); fD = 148–150; Ti I 206–212; Ti III 251–264.

**Description.** Dorsum. Idiosoma is almost oval (the holotype was used for drawing and its posterior cuticle was broken during slide preparation), with 148 barbed setae (fD = 148–150 in paratypes) (Figure 6A). Scutum length slightly shorter than width, with two pairs of sensilla (ASE and PSE), and two pairs of scutalae (AL and PL); anterior margin concave, anterolateral margins slightly cambered, posterolateral margins sinuous, posterior margin somewhat convex (Figure 7A and Figure 8). ASE bases are slightly posterior to the level of AL bases, PSE near the posterior margin of the scutum. ASE, PSE, AL and PL are all entirely barbed, and PSE much longer than ASE, AL and PL subequal (Table 2).

Venter. All ventral setae, including coxalae, barbed and acute (Figure 6B). Two intercoxal setae present between coxae I (*1a*) and between coxae II (*2a*), respectively. Four intercoxal setae (*3a_1_* and *3a_2_*) located between coxae II and III, *3a_1_* distinctly shorter than *3a_2_*, *1a* longer than *2a*, and *2a* subequal to *3a_2_* (Table 2). Three pairs of coxalae (*1b*, *2b* and *3b*) present, *1b* much longer than *2b* and *3b*, *3b* longer than *2b*.

Gnathosoma (Figure 7B). With two nude galealae (*cs*), two nude hypostomalae (*bs*), *bs* longer than *cs*. Palpfemur and palpgenu with one barbed seta on the dorsal surface (PaScFed and PaScGed), respectively. Palptibia with two barbed setae and one nude seta, odontus simple. Palptarsus with seven setae, of which, five nude setae, one solenidion (ω) and one eupathidium (ζ). fPp = 0-B-B-2BN-5Nωζ. Palpal supracoxal seta (*elcp*) peg-like.

Legs (Figure 9 and Figure 10). With seven segments (femora divided). IP = 2548–2560 (Holotyp and two paratypes). Dorsum of coxa I with a supracoxal seta (*eI*) which is peg-like and apically rounded. Leg setal formula: leg I: Cx—1n; Tr—1n; Bfe—2n; Tfe—5n; Ge—1σ, 1κ, 8n; Ti—2φ, 1κ, 14n; Ta—1ω, 1ε, 2ζ, 23n. leg II: Cx—1n; Tr—1n; Bfe—2n; Tfe—5n; Ge—1κ, 8n; Ti—2φ, 14n; Ta—1ω, 2ζ, 20n. leg III: Cx—1n; Tr—1n; Bfe—1n; Tfe—5n; Ge—8n; Ti—1φ, 15n; Ta—1ζ, 22n. The lengths of the legs were measured from coxae to tarsus (Table 2).

**Etymology.** The name of the new species is derived from Bomi where it was collected.

**Types.** Holotype, larva, from an unidentified moth (Lepidoptera), collected by Si-yuan Xu on 17 July 2019, from Guxiang Town (Altitude: 2673 m), Bomi County, Tibet Autonomous Region, China. Paratypes: one larva, an unidentified Elateridae (Coleoptera), collected by Si-yuan Xu on 18 July 2019, from Bomi County (Altitude: 3084 m), Tibet Autonomous Region, China; one larva, an unidentified Pentatomidae (Hemiptera), collected by Si-yuan Xu on 20 July 2019, from Bomi County (Altitude: 3374 m), Tibet Autonomous Region, China.

The holotype and paratypes are deposited in the Institute of Entomology, Guizhou University, Guiyang, China (GUGC).

**Distribution.** China: Tibet Autonomous Region.

**Remarks.** *Leptus* (*Leptus*) *bomiensis* **sp. nov.** keyed to the *phalangii* species group and *killingtoni* species subgroup proposed by Saboori et al. [16]. The *killingtoni* species subgroup consists of 12 species, of which, *L.* (*L.*) *albertensis* Southcott, 1992 was from Canada [31]; *L.* (*L.*) *brachypodos* Zheng, 1996, *L.* (*L.*) *dolichopodos* Zheng, 1996, *L.* (*L.*) *shimenensis* Zheng, 1996 and *L.* (*L.*) *sulciscutus* Zheng, 1996 from China [36]; *L.* (*L.*) *cavernicola* Fain and Elsen, 1987 from Rwanda [30]; *L.* (*L.*) *droozi* Southcott, 1992 from the United States of America [31]; *L.* (*L.*) *grossi* Southcott, 1999 from Australia [29]; *L.* (*L.*) *killingtoni* Turk, 1945 from Portugal (Azores Islands), Spain and the United Kingdom [31]; *L.* (*L.*) *scutellatus* Southcott, 1999 from Papua New Guinea [29]; *L.* (*L.*) *singhi* Saboori and Arbabi, 2003 from India [55]; *L.* (*L.*) *ubudicus* Haitlinger, 2006 from Indonesia (Lesser Sunda Islands) [56].

Saboori et al. [16] placed *L.* (*L.*) *dolichopodos* in the *phalangii* species group and *killingtoni* species subgroup. However, the original description there were two solenidia (σ) on Ge I (Seventh line of page 239 and Figures 27–32) [36] suggesting it should be grouped into the *torresianus* species subgroup.

In this paper, we still compare the new species with *L.* (*L.*) *dolichopodos*.

*Leptus* (*L*.) *bomiensis* **sp. nov.** differs from *L.* (*L.*) *albertensis* by the number of setae in fD formula (148–150 vs. 103–105); the shape of *3a*_1_ (pointed end vs. bifid); longer L (112–123 vs. 102), W (129–135 vs. 105), PW (114–122 vs. 97), PSE (87–94 vs. 68), Ti I (206–212 vs. 187), and Ti III (251–264 vs. 231). 

*Leptus* (*L*.) *bomiensis* **sp. nov.** differs from *L.* (*L.*) *brachypodos* by the number of setae in fD formula (148–150 vs. 54); longer L (112–123 vs. 98), W (129–135 vs. 111), AL (84–87 vs. 52), PL (84–89 vs. 66), Ti I (206–212 vs. 135), Ti II (176–190 vs. 123), and Ti III (251–264 vs. 162).

*Leptus* (*L*.) *bomiensis* **sp. nov.** differs from *L.* (*L.*) *cavernicola* by the number of setae in fD formula (148–150 vs. 50); longer L (112–123 vs. 84), W (129–135 vs. 78), AL (84–87 vs. 45), PL (84–89 vs. 55–60), PSE (87–94 vs. 58), Ti I (206–212 vs. 102), Ti III (251–264 vs. 142), and Ta I (168–174 vs. 95).

*Leptus* (*L*.) *bomiensis* **sp. nov.** differs from *L.* (*L.*) *dolichopodos* by the number of setae in fD formula (148–150 vs. 68), the number of solenidia on Ge I (1 vs. 2); the shorter Ti I (206–212 vs. 250) and Ti III (251–264 vs. 320).

*Leptus* (*L*.) *bomiensis* **sp. nov.** differs from *L.* (*L.*) *droozi* by the number of setae in fD formula (148–150 vs. 79); the longer L (112–123 vs. 87), W (129–135 vs. 87), Ti I (206–212 vs. 176), Ti II (176–190 vs. 151), Ti III (251–264 vs. 205), Leg I (860–868 vs. 730), and Leg III (922–934 vs. 740).

*Leptus* (*L*.) *bomiensis* **sp. nov.** differs from *L.* (*L.*) *grossi* by the longer Ti I (206–212 vs. 127), Ti II (176–190 vs. 111), Ti III (251–264 vs. 145), Leg I (860–868 vs. 550), Leg II (758–763 vs. 520), Leg III (922–934 vs. 620), L (112–123 vs. 86), W (129–135 vs. 95), AL (84–87 vs. 62), PL (84–89 vs. 64).

*Leptus* (*L*.) *bomiensis* **sp. nov.** differs from *L.* (*L.*) *killingtoni* by the longer Ti I (206–212 vs. 134–160), Ti II (176–190 vs. 110–136), Ti III (251–264 vs. 158–182), Ta I (168–174 vs. 112–132), Ta II (141–151 vs. 104–124), Ta III (170–180 vs. 110–129), Leg I (860–868 vs. 604–682), Leg II (758–763 vs. 554–650), Leg III (922–934 vs. 642–735), L (112–123 vs. 80–104), W (129–135 vs. 96–114).

*Leptus* (*L*.) *bomiensis* **sp. nov.** differs from *L.* (*L.*) *scutellatus* as follows: the dorsal of the cheliceral bases with longitudinal striations (no vs. yes); gnathosoma venter with fine transverse striations (no vs. yes); the number of setae in fD formula (148–150 vs. 46); longer Ti I (206–212 vs. 119), Ti III (251–264 vs. 140), L (112–123 vs. 69), W (129–135 vs. 76), ASE (51–54 vs. 29), and PSE (87–94 vs. 48).

*Leptus* (*L*.) *bomiensis* **sp. nov.** differs from *L.* (*L.*) *shimenensis* by the number of setae in fD formula (148–150 vs. 54) and fV (42–44 vs. 20); longer L (112–123 vs. 85), W (129–135 vs. 82), AW (99–103 vs. 62), PW (114–122 vs. 75), AL (84–87 vs. 68), and PL (84–89 vs. 65).

*Leptus* (*L*.) *bomiensis* **sp. nov.** differs from *L.* (*L.*) *singhi* by the number of setae in fD formula (148–150 vs. 60); longer Ti I (206–212 vs. 153), Ti II (176–190 VS. 138), Ti III (251–264 vs. 187), L (112–123 vs. 78), W (129–135 vs. 87), ASE (51–54 vs. 36), PSE (87–94 vs. 51), AL (84–87 vs. 56), and PL (84–89 vs. 68).

*Leptus* (*L*.) *bomiensis* **sp. nov.** differs from *L.* (*L.*) *sulciscutus* by the number of setae in fD formula (148–150 vs. 59) and fV (42–44 vs. 23); microseta on Ge II (present vs. absent); longer L (112–123 vs. 85), W (129–135 vs. 95), Leg I (860–868 vs. 800), and Leg III (922–934 vs. 840).

*Leptus* (*L*.) *bomiensis* **sp. nov.** differs from *L.* (*L.*) *ubudicus* by the one half of gnathosoma ventral surface with transverse striations (no vs. yes); the number of setae in fD formula (148–150 vs. 52), fV (42–44 vs. 16); longer Ti I (206–212 vs. 70), Ti II (176–190 vs. 64), and Ti III (251–264 vs. 92).

**Figure 11:**
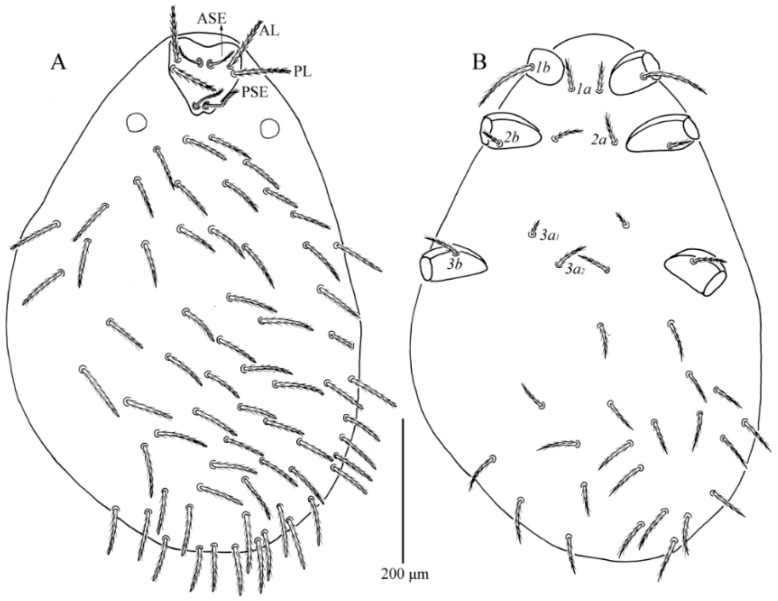
*Leptus (Leptus) longisolenidionus* **sp. nov.**, larva. (**A**). Dorsal view of idiosoma. (**B**). Ventral view of idiosoma.

**Figure 12:**
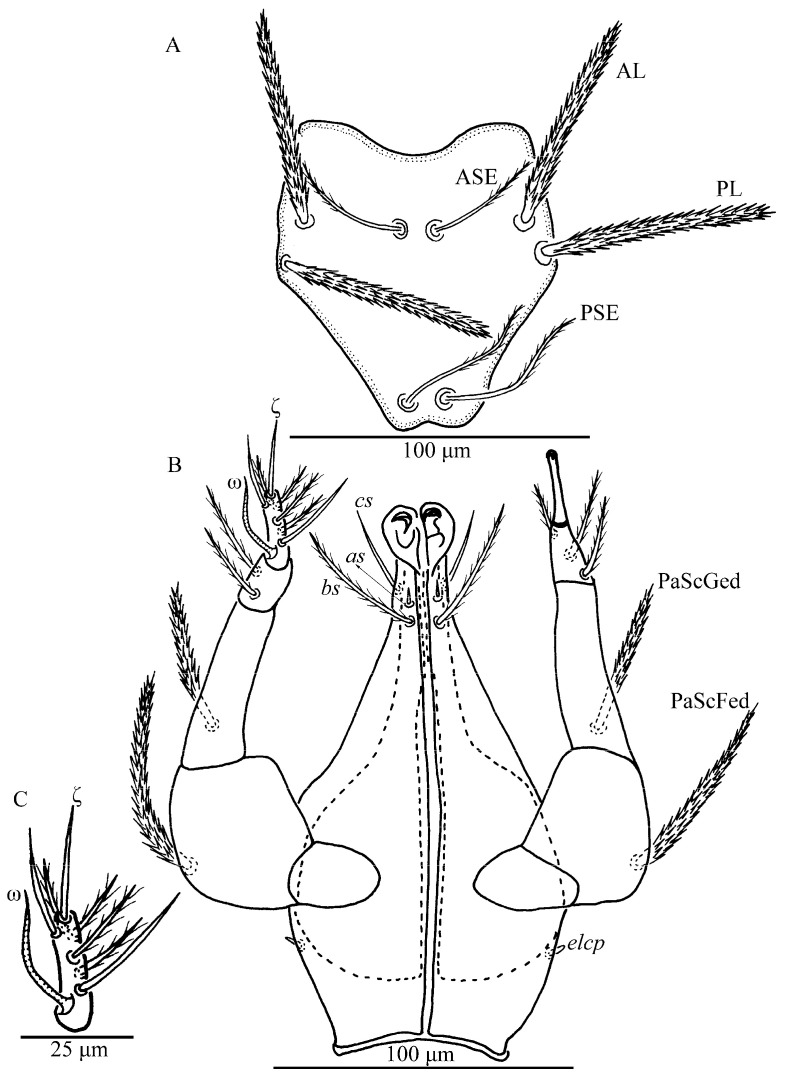
*Leptus (Leptus) longisolenidionus* **sp. nov.**, larva. (**A**). Scutum. (**B**). Ventral view of gnathosoma. (**C**). Ventral view of palptarsus.

**Figure 13:**
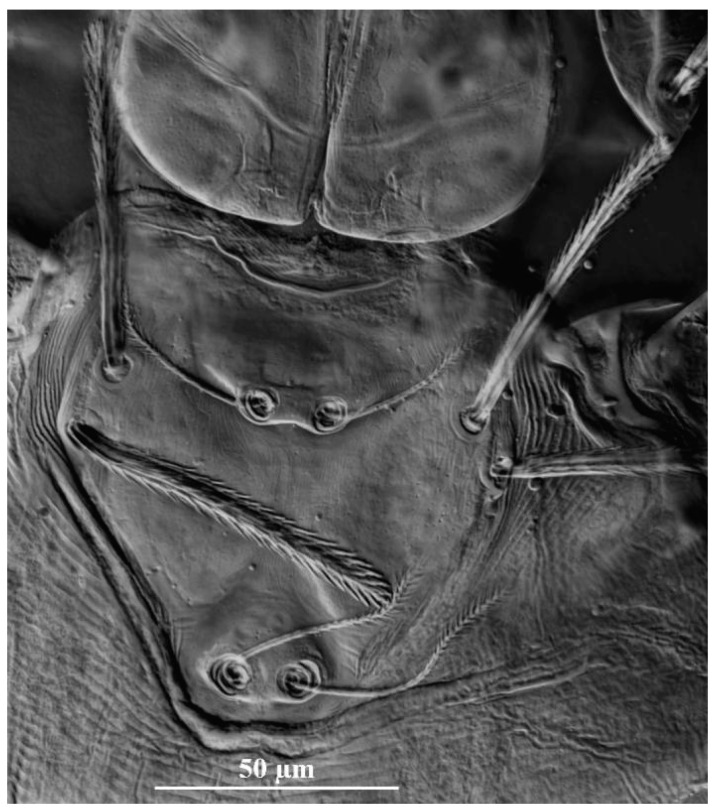
*Leptus (Leptus) longisolenidionus* **sp. nov.**, larva. Showing ASE and PSE shape and outline of scutum.

**Figure 14:**
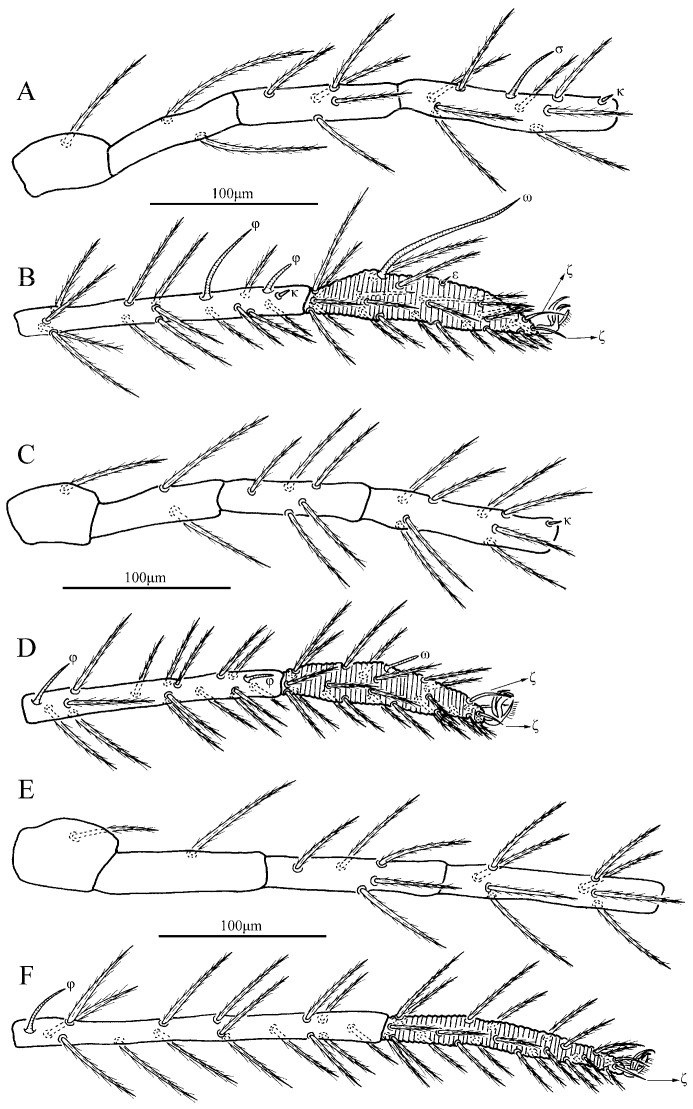
*Leptus (Leptus) longisolenidionus* **sp. nov.**, larva. (**A**). Leg I, trochanter—genu. (**B**). Leg I, tibia—tarsus. (**C**). Leg II, trochanter—genu. (**D**). Leg II, tibia—tarsus. (**E**). Leg III, trochanter—genu. (**F**). Leg III, tibia—tarsus.

**Figure 15:**
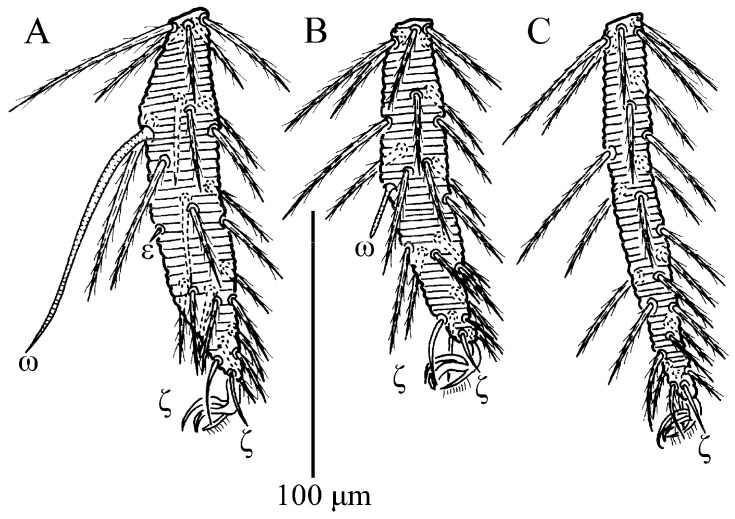
*Leptus (Leptus) longisolenidionus* **sp. nov.**, larva. (**A**). Leg I, tarsus. (**B**). Leg II, tarsus. (**C**). Leg III, tarsus.

**Figure 16:**
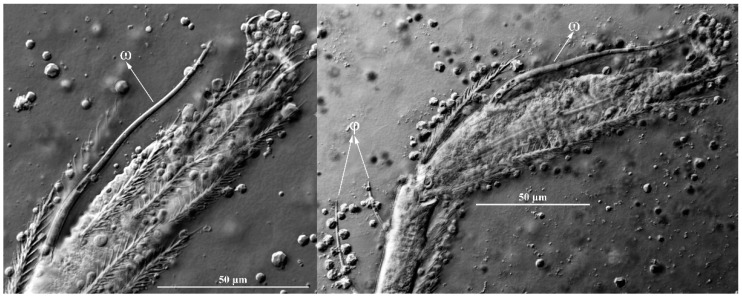
*Leptus (Leptus) longisolenidionus* **sp. nov.**, larva. Showing ωI on TaI.

*Leptus (Leptus) longisolenidionus* Xu and Jin **sp. nov.** (Figure 11, Figure 12, Figure 13, Figure 14, Figure 15 and Figure 16)

**Diagnosis (larva).** All normal setae of Ta I shorter than ωI, ωI > 90; ASE and PSE with fine barbs on distal halves; ASE posterior to AL; gnathosoma with four hypostomalae (*as* and *bs*); palpfemur and palpgenu each with one barbed seta on the dorsal surface (PaScFed and PaScGed); fD = 64–68; Ti I 163–171; Ti III 209–219; Ti III/AW 2.73–3.03.

**Description.** Dorsum. Idiosoma with 64 barbed setae (fD = 64–68 in paratypes) (Figure 11A). Scutum with two pairs of sensilla (ASE and PSE), and two pairs of scutalae (AL and PL), anterior margin concave in middle, anterolateral margins almost straight, posterolateral margins slightly concave, posterior margin concave between the bases of PSE (Figure 12A and Figure 13). ASE posterior to the level of AL, PSE near posterior margin of scutum; approximately distal half of ASE and PSE with fine barbs (Figure 12A and Figure 13); PSE longer than ASE; PL slightly longer than AL, both entirely barbed (Table 3).

Venter. All ventral setae, including coxalae, barbed and acute (Figure 11B). Two pairs of intercoxal setae (*1a* and *2a*), *1a* posterior to level of the posterior edge of coxae I, *2a* between coxae II. Four intercoxal setae (*3a_1_* and *3a_2_*) present between coxae II and III, *3a_2_* distinctly longer than *3a_1_*, *1a*, *2a* and *3a_2_* almost the same length. Three pairs of coxalae (*1b*, *2b* and *3b*), *1b* much longer than *2b* and *3b*, *3b* longer than *2b* (Table 3). 22 setae behind coxae III (fV = 22–24 in paratypes).

Gnathosoma (Figure 12B). With two nude galealae (*cs*), four hypostomalae (*as* and *bs*), *as* nude, pointed and minute, *bs* barbed and pointed; *bs* much longer than *as*, *bs* longer than *cs*. Palpfemur and palpgenu each with one barbed dorsal seta (PaScFed and PaScGed). Palptibia with three barbed setae, one of them on the ventral surface, odontus simple. Palptarsus with eight setae, of which four barbed, two nude, one solenidion (ω) and one eupathidium (ζ). fPp = 0-B-B-3B-4B2Nωζ. Palpal supracoxal seta (*elcp*) peg-like.

Legs (Figure 14, Figure 15 and Figure 16). With seven segments (femora divided). IP = 2104–2189 (Holotyp and four paratypes). Solenidion of Ta I longer than all normal setae of Ta I, ωI > 90 (Figure 14B, Figure 15A and Figure 16). Dorsum of coxa I with a supracoxal seta (*eI*) which is peg-like and with a rounded tip. Leg setal formula: leg I: Cx—1n; Tr—1n; Bfe—2n; Tfe—5n; Ge—1σ, 1κ, 8n; Ti—2φ, 1κ, 14n; Ta—1ω, 1ε, 2ζ, 24n. leg II: Cx—1n; Tr—1n; Bfe—2n; Tfe—5n; Ge—1κ, 8n; Ti—2φ, 15n; Ta—1ω, 2ζ, 23n. leg III: Cx—1n; Tr—1n; Bfe—1n; Tfe—5n; Ge—8n; Ti—1φ, 15n; Ta—1ζ, 25n. The lengths of the legs were measured from coxae to tarsus (Table 3).

**Etymology.** The new species is named after a distinctly long solenidion on Ta I.

**Types.** Holotype, larva, from an unknown insect host, collected by Xin-feng Zhang on 25 April 2009, from Bawangling National Natural Reserve, Hainan Province, China. Paratypes: four larvae, the same data as the holotype.

The holotype and paratypes are deposited in the Institute of Entomology, Guizhou University, Guiyang, China (GUGC).

**Distribution.** China: Hainan Province.

**Remarks.** On the keys by Saboori et al. [16] *Leptus* (*L*.) *longisolenidionus* **sp. nov.** can be placed in the *phalangii* group and *phalangii* subgroup along with 27 other species. The similar species of *L*. (*L*.) *longisolenidionus* **sp. nov.,** sharing the characters Ti III/AW > 2, 200 < Ti III ≤ 235, L > 95, are *L*. (*L*.) *californicus* Southcott, 1992 [31], *L*. (*L*.) *holgeri* Haitlinger, 1999 [57], *L*. (*L*.) *nearcticus* Fain, Gummer and Whitaker, 1987 [58], *L*. (*L*.) *phalangii* (de Geer, 1778) [59], and *L*. (*L*.) *swani* Southcott, 1991 [26].

*Leptus* (*Leptus*) *longisolenidionus* **sp. nov.** differs from *L*. (*L*.) *californicus* by the number of hypostomalae (four setae vs. two setae), gnathosomal venter without striations (vs. with coarse transverse striations); longer ωI (92–99 vs. 27), DS (41–74 vs. 32–38), PDS (50–74 vs. 32–38), AL (69–74 vs. 42), PL (75–80 vs. 37), shorter W (91–97 vs. 115), PSE (46–54 vs. 80).

*Leptus* (*Leptus*) *longisolenidionus* **sp. nov.** differs from *L*. (*L*.) *holgeri* by the number of setae in fD formula (64–68 vs. 45), the number of hypostomalae (four vs. two); longer ωI (92–99 vs. 24–28); shorter L (96–104 vs. 118–130), W (91–97 vs. 120–130), PW (82–90 vs. 106–118), PSE (46–54 vs. 70–80), Leg I (689–699 vs. 726–802).

*Leptus* (*Leptus*) *longisolenidionus* **sp. nov.** differs from *L*. (*L*.) *nearcticus* by the number of setae in fD formula (64–68 vs. 94); longer ωI (92–99 vs. 33), Leg I (689–699 vs. 651), Leg II (631–670 vs. 625), Leg III (775–820 vs. 724); shorter ISD (50–59 vs. 87–100), fn Ti (14-15-15 vs. 14-16-16).

*Leptus* (*Leptus*) *longisolenidionus* **sp. nov.** differs from *L*. (*L*.) *phalangii* by the number of setae in fD formula (64–68 vs. 98), ωI much longer than all normal setae of Ta I (yes vs. no), shorter W (91–97 vs. 118–123), ASE (33–39 vs. 60), PSE (46–54 vs. 78), ISD (50–59 vs. 70–75), PW (82–90 vs. 103–113), Ti I (163–171 vs. 203–213), Ti II (142–152 vs. 170–185), Ti III (209–219 vs. 233–235), IP (2104–2189 vs. 2633).

*Leptus* (*Leptus*) *longisolenidionus* **sp. nov.** differs from *L*. (*L*.) *swani* by the number of hypostomalae (4 vs. 2); longer ωI (92–99 vs. 26), PDS (50–74 vs. 33–36), PL (75–80 vs. 50); shorter L (96–104 vs. 118), W (91–97 vs. 121), AW (69–79 vs. 93), PW (82–90 vs. 113), PSE (46–54 vs. 72), Leg I (689–699 vs. 820), Leg II (631–670 vs. 715), Leg III (775–820 vs. 855).

**Figure 17:**
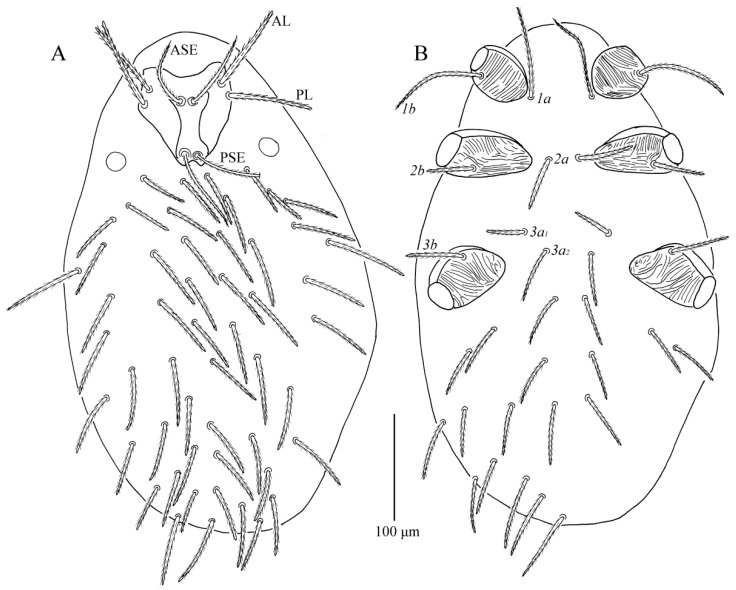
*Leptus (Leptus) striatus* **sp. nov.**, larva. (**A**). Dorsal view of idiosoma. (**B**). Ventral view of idiosoma.

**Figure 18:**
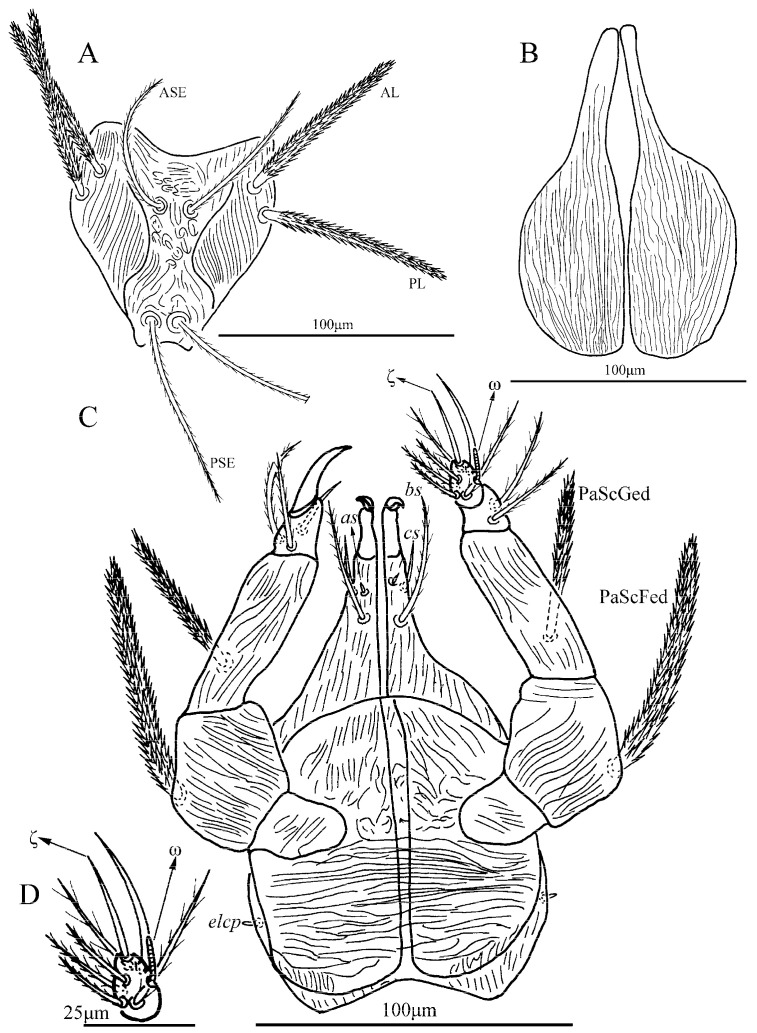
*Leptus (Leptus) striatus* **sp. nov.**, larva. (**A**). Scutum. (**B**). Dorsal view of the cheliceral base. (**C**). Ventral view of gnathosoma. (**D**). Ventral view of palptarsus.

**Figure 19:**
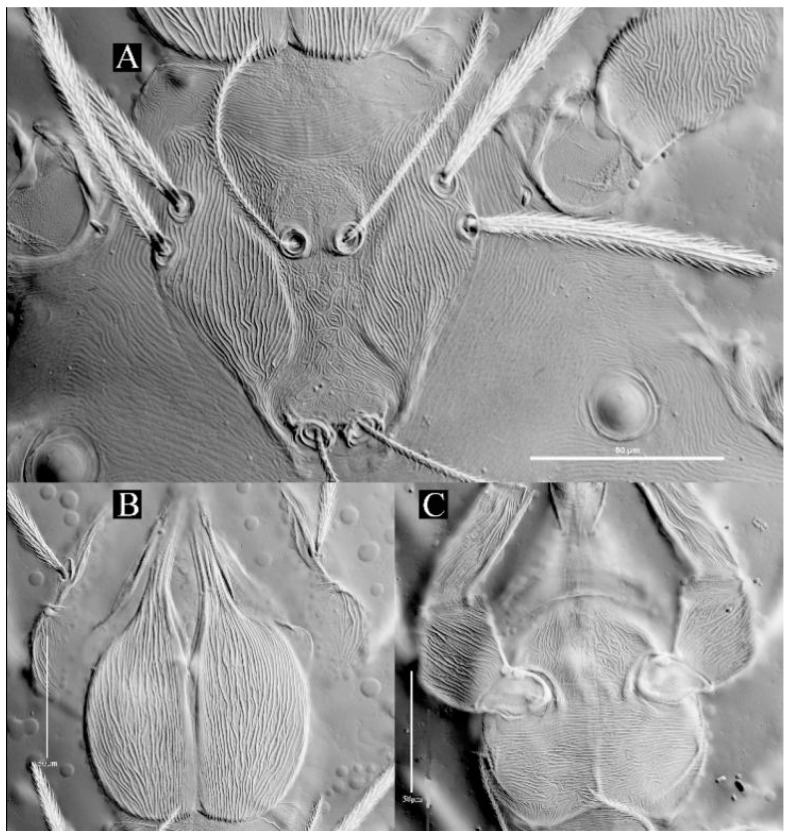
*Leptus (Leptus) striatus* **sp. nov.**, larva. (**A**). Scutum. (**B**). Dorsal view of the cheliceral base. (**C**). Ventral view of basis capitula, palpfemur and palpgenu. Scale bar = 50 µm.

**Figure 20:**
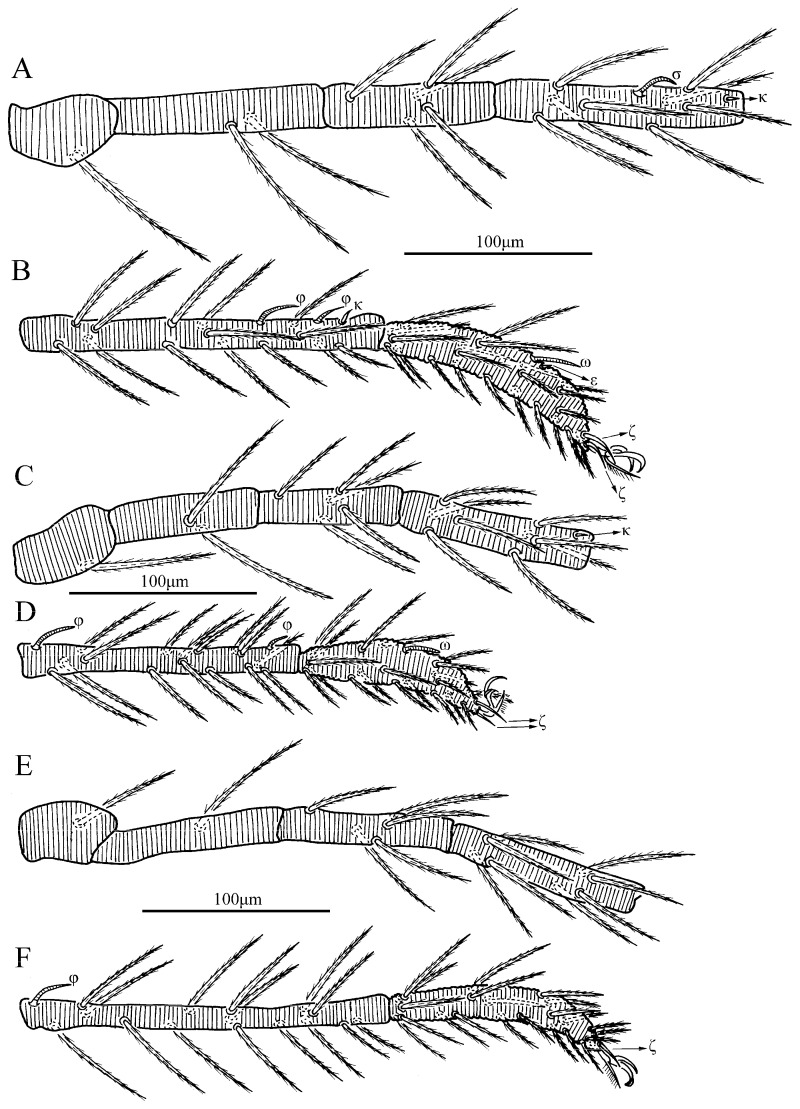
*Leptus (Leptus) striatus* **sp. nov.**, larva. (**A**). Leg I, trochanter—genu. (**B**). Leg I, tibia—tarsus. (**C**). Leg II, trochanter—genu. (**D**). Leg II, tibia—tarsus. (**E**). Leg III, trochanter—genu. (**F**). Leg III, tibia—tarsus.

**Figure 21:**
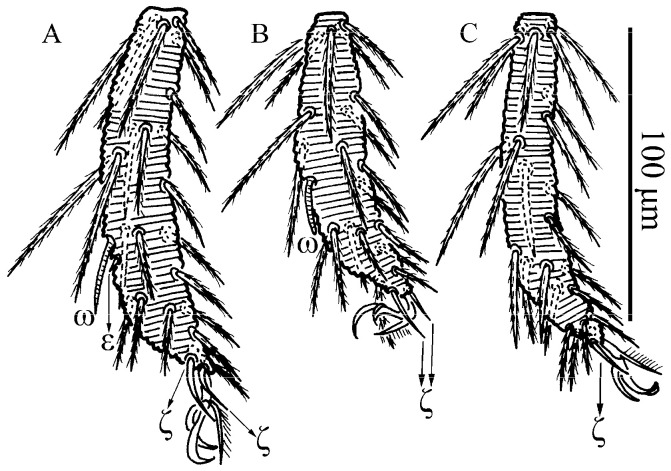
*Leptus (Leptus) striatus* **sp. nov.**, larva. (**A**). Leg I, tarsus. (**B**). Leg II, tarsus. (**C**). Leg III, tarsus.

*Leptus (Leptus) striatus* Xu and Jin **sp. nov.** (Figure 17, Figure 18, Figure 19, Figure 20 and Figure 21)

**Diagnosis (larva).** Cheliceral base dorsally with numerous longitudinal sinuous striations; venter of basis capituli proximally with transverse striations, and distally with numerous fine longitudinal striations; palpfemur and palpgenu with numerous fine striations, and each with one barbed seta on the dorsal surface (PaScFed and PaScGed); scutum with longitudinal striations on both sides, and small disordered striations in the median area; ventral view of coxae I, II and III with numerous fine striations; ASE and PSE entirely with fine barbs; gnathosoma with four hypostomalae; fD = 52–56; Ti I 177–203; Ti III 186–219.

**Description.** Dorsum. Idiosoma with 52–56 barbed setae (fD = 52 in holotype) (Figure 17A). Scutum length is slightly longer than the width (Table 4), the anterior margin is concave, anterolateral margins and posterolateral margins are slightly sinuous, posterior margin concave (Figure 18A); with longitudinal striations on both sides, and small disordered striations in median region (Figure 18A and Figure 19A); with two pairs of sensilla (ASE and PSE) and two pairs of scutalae (AL and PL); ASE located between AL and PL bases and almost at same level of PL; PSE near posterior margin of scutum; ASE and PSE with fine barbs throughout the length, PSE slightly longer than ASE; PL slightly longer than or subequal to AL, both entirely barbed (Table 4).

Venter. All ventral setae, including coxalae, barbed and acute (Figure 17B). Coxae I, II and III ventrally with numerous fine striations (Figure 17B). Two barbed intercoxal setae present between coxae I (*1a*) and between coxae II (*2a*), respectively. Four intercoxal setae (*3a_1_* and *3a_2_*) between coxae III with *3a_1_* somewhat anteriorly located; *3a_2_* distinctly longer than *3a_1_*, *1a* longer than *2a*, *2a* and *3a_2_* subequal (Table 4). Three pairs of coxalae (*1b*, *2b* and *3b*), *1b* much longer than *2b* and *3b*, *2b* and *3b* subequal, *1b* and *1a* almost subequal (Table 4). Area behind coxae III with 18–20 setae (fV = 18 in holotype).

Gnathosoma. With one pair of nude galealae (*cs*), two pairs of hypostomalae (*as* and *bs*), *as* nude and *bs* barbed (Figure 18); *bs* much longer than *as*, *bs* longer than *cs* (Table 4). Palpfemur and palpgenu with numerous fine striations, and each with one barbed seta on dorsal surface (PaScFed and PaScGed) (Figure 18C and Figure 19C). Palptibia with two barbed setae and one nude seta, ventral surface with one barbed seta, odontus simple. Palptarsus with eight setae, of which five barbed, one nude, one solenidion (ω) and one eupathidium (ζ) (Figure 18D). fPp = 0-B-B-2BN-5BNωζ. Cheliceral base dorsally with numerous longitudinal sinuous striations; ventral of basis capituli proximally with transverse striations and distally with numerous fine longitudinal striations (Figure 18B,C and Figure 19B,C). Palpal supracoxal seta (*elcp*) peg-like.

Legs (Figure 20 and Figure 21). With seven segments (femora divided). IP = 2038–2291 (holotyp and 17 paratypes). Dorsum of coxa I with a supracoxal seta (*eI*) which is peg-like with a rounded tip. Leg setal formula: leg I: C x—1n; Tr—1n; Bfe—2n; Tfe—5n; Ge—1σ, 1κ, 8n; Ti—2φ, 1κ, 14n; Ta—1ω, 1ε, 2ζ, 23n. leg II: Cx—1n; Tr—1n; Bfe—2n; Tfe—5n; Ge—1κ, 8n; Ti—2φ, 15n; Ta—1ω, 2ζ, 20n. leg III: Cx—1n; Tr—1n; Bfe—1n; Tfe—5n; Ge—8n; Ti—1φ, 15n; Ta—1ζ, 23n. The lengths of legs were measured from coxae to tarsus (Table 4).

**Etymology.** The new species is named after exclusively striated gnathosoma, scutum and coxae. 

**Types.** Holotype, larva, an unidentified Opiliones, collected by Si-yuan Xu on 7 May 2016, from Xishuangbanna National Natural Reserve (Altitude: 647 m), Yunnan Province, China. Paratypes: six larvae, the same data as the holotype; three larvae, an unidentified Opiliones, collected by Si-yuan Xu on 7 May 2016, from Xishuangbanna National Natural Reserve (Altitude: 647 m); five larvae, an unidentified Opiliones, collected by Si-yuan Xu on 10 May 2016, from Xishuangbanna National Natural Reserve (Altitude: 672 m); one larva, an unidentified Opiliones, collected by Xue-song Zhang on 25 April 2018, from Xishuangbanna National Natural Reserve (Altitude: 633 m); two larvae, an unidentified Opiliones, collected by Si-yuan Xu on 12 November 2018 from Xishuangbanna National Natural Reserve (Altitude: 1023 m), Yunnan Province, China.

The holotype and paratypes are deposited in the Institute of Entomology, Guizhou University, Guiyang, China (GUGC).

**Distribution.** China: Yunnan Province.

**Remarks.** *Leptus* (*Leptus*) *striatus* **sp. nov.** and *L.* (*L.*) *bomiensis* **sp. nov.** belongs to the *killingtoni* subgroup of the *phalangii* species group. 

*Leptus* (*Leptus*) *striatus* **sp. nov.** can be easily separated from *L.* (*L.*) *albertensis*, *L.* (*L.*) *grossi*, *L.* (*L.*) *killingtoni*, and *L.* (*L.*) *bomiensis* **sp. nov.**, based on fD = 52–56 in *L.* (*L.*) *striatus* **sp. nov.** (vs. fD > 100 in the later four species); *L.* (*L.*) *striatus* **sp. nov.** can also be separated from *L.* (*L.*) *brachypodos*, *L.* (*L.*) *dolichopodos*, *L.* (*L.*) *cavernicola*, *L.* (*L.*) *droozi*, *L.* (*L.*) *shimenensis*, *L.* (*L.*) *singhi*, and *L.* (*L.*) *sulciscutus*, based on the strated cheliceral base, palpfemur, palpgenu, scutum and venter of coxae I, II, and III in *L.* (*L.*) *striatus* **sp. nov.** (vs. without striations according to the original descriptions and illustrations of the seven species above).

In the *killingtoni* subgroup, there are only two species with striations on gnathosoma, one is *L*. (*L*.) *scutellatus* (dorsum of the chelicera with longitudinal striations and basis capitula with transverse striations), another one is *L*. (*L*.) *ubudicus* (gnathosomal venter half striated). 

*Leptus* (*Leptus*) *striatus* **sp. nov.** differs from *L.* (*L.*) *scutellatus* as follows: proximal venter of basis capitula with transverse striations, and distal venter of basis capitula with longitudinal striations in *L.* (*L.*) *striatus* **sp. nov.** (vs. proximal basis capitula with transverse striations in *L.* (*L.*) *scutellatus*); scutum, palpfemur, and palpgenu with striations in *L.* (*L.*) *striatus* **sp. nov.** (vs. absent in *L.* (*L.*) *scutellatus*); longer Ti I (177–203 vs. 119), Ti II (139–160 vs. 102), Ti III (186–219 vs. 140).

*Leptus* (*Leptus*) *striatus* **sp. nov.** differs from *L.* (*L.*) *ubudicus* by dosum of the cheliceral base with striations (vs. without striations in *L.* (*L.*) *ubudicus*); scutum, palpfemur and palpgenu with striations (vs. absent in *L.* (*L.*) *ubudicus*); longer Ti I (177–203 vs. 70), Ti II (139–160 vs. 64), Ti III (186–219 vs. 92).

### 3.2. Checklist and Distribution of Charletonia and Leptus from China 

*Charletonia banksi* Southcott, 1966

*C*. *hunanensis* Zheng, 1996: 65. Synonymized by Hakmitabar and Saboori [1].

Type locality: nine miles (about 14.48 km) ESE of Capella, Queensland, Australia.

Host. Unidentified Libellulidae (Insecta: Odonata) and unidentified

Megapodagriidae (Insecta: Odonata) in China; *Goniaea vocans*, *Hepalieus gracilis*, *Nomaducris guttulosa* and *Oedaleus australis* in Australia.

Distribution. Australia, China (Hunan Province).

*Charletonia taiwanensis* Tsai and Chow, 1988

Type locality. The mountainous area around Taipei (Taibei) city, Taiwan Province, China.

Host. *Chondracris rosea* (Insecta: Orthoptera: Acrididae).

Distribution. China (Taiwan Province).

*Leptus (Leptus) astrubali* Haitlinger, 1999

*L*. (*L*.) *coloanensis* Haitlinger, 2006: 91. Synonymized by Saboori et al. [16].

Type locality. Ayutthaya, Thailand.

Host. Unknown. This species was collected only from herbaceous plants. 

Distribution. China (Macao Special Administrative Region), India, Myanmar, Nepal, Thailand.

*Leptus (Leptus) brachypodos* Zheng, 1996

Type locality. Mt. Hupingshan, Shimen County, Hunan Province, China.

Host. Unknown.

Distribution. China (Hunan Province).

*Leptus (Leptus) dolichopodos* Zheng, 1996

Type locality. Mt. Hupingshan, Shimen County, Hunan Province, China.

Host. Unknown.

Distribution. China (Hunan Province).

*Leptus (Leptus) guilinicus* Haitlinger, 2006

Type locality. Yangshou County, Guilin City, Guangxi Province, China.

Host. Unknown. This species was collected from herbaceous plants.

Distribution. China (Guangxi Province). 

*Leptus (Leptus) hupingshanicus* Zheng, 1996

Type locality. Mt. Hupingshan, Shimen County, Hunan Province, China.

Host. Unknown.

Distribution. China (Hunan Province).

*Leptus (Leptus) shimenensis* Zheng, 1996

Type locality. Mt. Hupingshan, Shimen County, Hunan Province, China.

Host. Unknown.

Distribution. China (Hunan Province).

*Leptus (Leptus) siemsseni* (Oudemans, 1910)

Type locality. Futschou (Fuzhou?), Fokien (Fujian Province), China.

Host. Unknown.

Distribution. China (Fujian Province).

*Leptus (Leptus) sulciscutus* Zheng, 1996

Type locality. Mt. Hupingshan, Shimen County, Hunan Province, China.

Host. Unknown.

Distribution. China (Hunan Province).

*Leptus (Leptus) trisolenidionus* Xu and Jin, 2022

Type locality. Datian National Natural Reserve, Hainan Province, China.

Host. Unidentified Cicadellinae (Insecta: Hemiptera: Cicadellidae).

Distribution. China (Guizhou and Hainan Province).

*Leptus (Leptus) zhejiangensis* Zheng, 2003

Type locality. Mt. Tianmushan, Linan District, Hangzhou City, Zhejiang Province, China.

Host. *Siobla ferox* (Insecta: Hymenoptera: Tenthredinidae)

Distribution. China (Zhejiang Province).

*Leptus (Leptus) zhutingensis* Zheng, 1996

Type locality. Zhuting Town, Zhuzhou County, Hunan Province, China.

Host. *Colaspoides opaca* (Insecta: Coleoptera: Chrysomelidae) and unidentified Cicadidae (Insecta: Hemiptera: Cicadidae)

Distribution. China (Hunan Province).

Distribution data and host information were obtained from Haitlinger [38,60], Tasi and Chow [14], Xu et al. [40], Zhang [39], and Zheng [13,36,37].

## 4. Discussion

**Figure 22:**
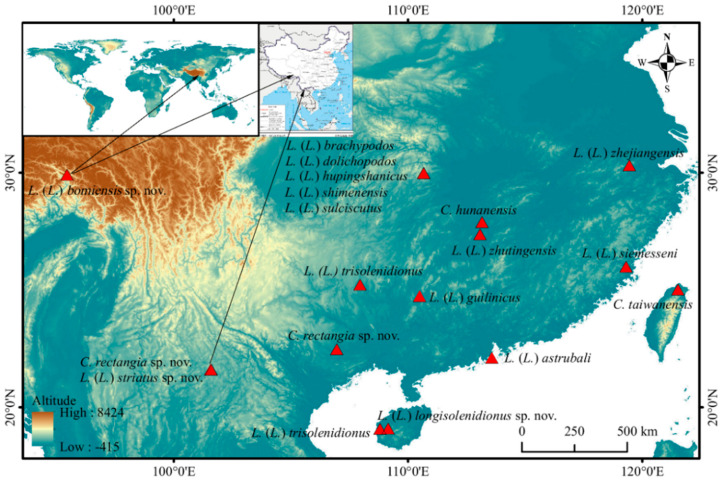
Distribution map of known and new species of the genera *Charletonia* and *Leptus* from China.

The genera *Charletonia* and *Leptus* are distributed worldwide (except Antarctica) with 86 and more than 240 species described with larvae, respectively. However, only three species of *Charletonia* and 14 species of *Leptus* were reported from China (Figure 22). *Charletonia* and *Leptus* in China represent less than 4% and 6% of the known world species, respectively. It suggests an urgent need to collect and study these genera and even the Erythraeidae in China.

Host records for Chinese Erythraeidae are mostly unknown. The literature and the current study recorded seven families in four insect orders and only one arachnid family for *Leptus*. For *Charletonia* hosts, only eight families in six insect orders are known in China, while there are 35 families in 11 insect orders and four arachnid orders reported worldwide.

Being ectoparasitic mites, the host information of larval Erythraeidae could be helpful for researchers to collect the mite specimens according to the host habits. The information of the hosts is quite important and should be recorded as detailed as possible when investigating the diversity of Erythraeid mites. In general, the comprehensive host information could provide valuable biological information for the study of erythraeid fauna, a phylogeny of the family, and co-evolution with the hosts.

## Figures and Tables

**Table 1:** Measurements of *Charletonia rectangia* **sp. nov.** (larvae, a–g = paratypes).

Character	Holotype	a	b	c	d	e	f	g	SD	Range
FD	54	54	58	55	54	56	54	54	1.36	54–58
FV	25	24	24	24	26	24	26	26	0.93	24–26
NDV	79	78	82	79	80	80	80	80	1.09	78–82
IL	593	451	308	1199	953	875	627	537	274.37	308–1199
IW	381	296	239	1001	564	576	433	384	223.81	239–1001
DS	39–71	38–67	43–66	41–69	42–69	43–74	41–72	38–66	1.93–2.73	38–74
PDS	44–71	46–64	49–66	47–69	51–69	49–71	47–72	44–65	2.32–2.83	44–72
Oc	23	26	24	24	28	25	23	27	1.73	23–28
*1a*	41	52	45	44	39	41	44	47	3.82	39–52
*1b*	117	118	115	116	119	117	116	118	1.22	115–119
*2a*	71	77	73	75	77	75	79	76	2.34	71–79
*2b_1_*	97	91	94	101	102	91	104	93	4.82	91–104
*2b_2_*	54	56	55	52	56	57	55	53	1.56	52–57
*3a*	67	63	63	66	69	70	69	72	3.04	63–72
*3b_1_*	78	77	81	84	85	78	83	79	2.87	77–85
*3b_2_*	53	52	51	52	55	50	56	51	1.94	50–56
L	100	105	109	107	100	102	95	103	4.15	95–109
W	133	131	134	139	140	141	134	129	4.11	121–141
AW	91	89	87	90	88	87	84	85	2.23	84–91
MW	111	108	102	106	107	107	104	104	2.62	102–111
PW	121	117	116	119	118	122	117	115	2.26	115–122
MA	42	36	38	37	37	40	36	37	1.96	36–40
AA	16	15	16	15	17	16	15	15	0.70	15–17
SB	24	20	24	23	20	23	21	22	1.54	20–24
ISD	58	67	70	66	63	64	63	64	3.28	58–70
AP	39	44	43	41	42	45	39	41	2.05	39–45
AL	89	80	94	96	88	88	80	87	5.36	80–96
ML	57	47	54	57	60	54	51	44	5.05	44–60
PL	69	69	71	73	79	68	64	66	4.31	64–79
ASE	60	71	64	61	68	63	67	60	3.80	60–71
PSE	86	99	97	85	93	89	92	83	5.39	83–99
PaScFed	99	89	93	94	103	97	91	87	4.99	87–103
PaScGed	60	51	56	58	52	49	52	50	3.74	49–60
*as*	18	16	17	20	20	18	17	19	1.36	16–20
*bs*	45	43	44	41	39	40	41	42	1.90	39–45
*cs*	32	36	32	36	35	29	31	30	2.55	29–36
GL	177	161	174	177	184	180	168	163	7.68	161–184
Ta I (H)	21	20	18	21	21	20	19	23	1.41	18–23
Ta I (L)	189	183	191	192	198	193	181	182	5.68	181–198
Ti I	246	256	263	257	265	258	242	241	8.70	241–265
Ge I	188	185	193	192	199	191	181	175	7.05	175–199
TFe I	108	99	111	106	114	109	107	100	4.79	99–114
BFe I	113	119	120	117	121	114	113	111	3.50	111–121
Tr I	68	55	63	64	61	66	59	62	3.80	55–68
Cx I	67	62	71	72	68	66	67	66	2.91	62–72
Ta II (H)	21	19	20	19	20	22	20	21	0.97	19–22
Ta II (L)	175	173	177	173	184	174	173	167	4.47	167–184
Ti II	219	222	223	227	231	222	220	219	3.92	219–231
Ge II	167	152	158	160	164	162	168	158	4.94	152–168
TFe II	97	94	96	96	103	94	95	93	2.92	93–103
BFe II	113	104	112	107	112	101	102	106	4.43	101–113
Tr II	60	55	56	61	56	57	57	60	2.11	55–61
Cx II	83	71	80	77	78	76	79	77	3.24	71–83
Ta III (H)	19	20	17	19	18	20	21	21	1.32	17–21
Ta III (L)	183	180	191	195	196	196	186	186	5.84	180–196
Ti III	308	311	312	319	331	328	311	305	8.86	305–331
Ge III	203	183	194	199	202	203	187	193	7.11	183–203
TFe III	130	132	138	131	128	136	131	126	3.67	126–138
BFe III	121	124	125	118	120	122	120	121	2.12	118–125
Tr III	63	70	67	62	58	60	73	65	4.74	58–73
Cx III	94	82	84	86	92	87	90	88	3.76	82–94
Leg I	979	959	1012	1000	1026	997	950	937	29.52	937–1026
Leg II	914	871	902	901	928	886	894	880	17.27	871–928
Leg III	1102	1082	1111	1110	1127	1132	1098	1084	16.95	1082–1132
IP	2995	2912	3025	3011	3081	3015	2942	2901	57.80	2901–3081
σ	25	33	27	29	28	30	31	26	2.50	25–33
φ’I	53	53	53	57	60	55	56	52	2.52	52–60
φ’’I	42	44	40	40	43	42	41	44	1.50	40–44
φ’–φ’’I	19	17	17	18	16	18	20	17	1.20	16–20
ωI	35	40	37	39	34	36	37	37	1.83	34–40
φ’II	20	21	20	22	21	22	21	19	0.97	19–22
φ’’II	41	47	41	46	46	43	43	41	2.35	41–47
φ’–φ’’II	173	181	179	182	184	177	179	176	3.30	173–184
ωII	21	24	23	23	22	20	21	22	1.22	20–24
φ’III	46	51	47	47	50	47	45	49	1.92	45–51

**Table 2:** Measurements of *Leptus (Leptus) bomiensis* **sp. nov.** (larvae, a and b = paratypes).

Character	Holotype	a	b	SD	Range	Character	Holotype	a	b	SD	Range
FD	148	148	150	0.94	148–150	Ti I	212	206	211	2.62	206–212
FV	42	42	44	0.94	42–44	Ge I	139	146	143	2.87	139–146
NDV	190	190	194	1.89	190–194	TFe I	107	105	106	0.82	105–107
IL	684	702	843	71.09	684–843	BFe I	108	114	106	3.40	106–114
IW	420	453	532	46.99	420–532	Tr I	54	50	57	2.87	50–57
DS	50–74	47–72	53–76	1.63–2.45	47–76	Cx I	78	73	69	3.68	69–78
PDS	59–74	56–72	61–76	1.63–2.05	56–76	Ta II (H)	24	24	28	1.89	24–28
Oc	20	22	20	0.94	20–22	Ta II (L)	141	151	143	4.32	141–151
*1a*	62	57	54	3.30	54–62	Ti II	180	176	194	7.72	176–190
*1b*	99	85	91	5.73	85–99	Ge II	114	114	109	2.36	109–114
*2a*	47	48	50	1.25	47–50	TFe II	96	89	94	2.94	89–96
*2b*	40	37	39	1.25	37–40	BFe II	90	86	90	1.89	86–90
*3a_1_*	33	36	27	3.74	27–36	Tr II	55	55	52	1.41	52–55
*3a_2_*	47	45	44	1.25	44–47	Cx II	83	87	81	2.49	81–87
*3b*	61	57	56	2.16	56–61	Ta III (H)	21	20	22	0.82	20–22
L	123	112	117	4.50	112–123	Ta III (L)	173	170	180	4.19	170–180
W	132	129	135	2.45	129–135	Ti III	251	264	262	5.72	251–264
AW	99	103	101	1.63	99–103	Ge III	128	135	127	3.56	127–135
PW	114	122	121	3.56	114–122	TFe III	112	110	106	2.49	106–112
MA	43	45	44	0.82	43–45	BFe III	108	103	103	2.36	103–108
AA	15	16	16	0.47	15–16	Tr III	54	53	58	2.16	53–58
SB	21	22	21	0.47	21–22	Cx III	96	99	95	1.70	95–99
ISD	68	72	67	2.16	67–72	Leg I	867	868	860	3.56	860–868
AP	16	22	20	2.49	16–22	Leg II	759	758	763	2.16	758–763
AL	87	84	86	1.25	84–87	Leg III	922	934	931	5.10	922–934
PL	89	84	85	2.16	84–89	IP	2548	2560	2554	4.90	2548–2560
ASE	54	51	53	1.25	51–54	σ	29	32	31	1.25	29–32
PSE	91	87	94	2.87	87–94	φ’I	28	30	29	0.82	28–30
PaScFed	72	68	73	2.16	68–73	φ’’I	29	29	27	0.94	27–29
PaScGed	57	51	59	3.40	51–59	φ’–φ’’I	32	32	36	1.89	32–36
*bs*	51	50	53	1.25	50–53	ωI	33	34	38	2.16	33–38
*cs*	29	29	32	1.41	29–32	φ’II	25	29	26	1.70	25–29
GL	217	226	209	6.94	209–226	φ’’II	24	27	26	1.25	24–27
Ta I (H)	27	23	29	2.49	23–29	φ’–φ’’II	139	141	147	3.40	139–147
Ta I (L)	169	174	168	2.62	168–174	ωII	22	25	27	2.05	22–27
						φ’III	25	26	30	2.16	25–30

**Table 3:** Measurements of *Leptus (Leptus) longisolenidionus* **sp. nov.** (larvae, a–d = paratypes).

Character	Holotype	a	b	c	d	SD	Range	Character	Holotype	a	b	c	d	SD	Range
FD	64	64	68	64	66	1.60	64–68	Ti I	171	166	167	168	163	2.61	163–171
FV	22	22	24	22	22	0.80	22–24	Ge I	128	129	126	127	123	2.06	123–129
IL	723	601	653	262	413	169.07	262–723	TFe I	87	88	92	87	86	2.10	86–92
IW	492	400	434	169	299	113.75	169–492	BFe I	76	74	77	80	83	3.16	74–83
DS	49–74	46–73	41–74	47–70	51–68	2.40–3.37	41–74	Tr I	48	42	44	44	49	2.65	42–49
PDS	58–74	54–73	50–74	54–70	53–68	2.40–2.56	50–74	Cx I	53	56	51	61	57	3.44	51–61
Oc	18	18	21	16	20	1.74	16–21	Ta I (H)	23	26	19	22	24	2.32	19–26
*1a*	42	46	43	42	40	1.96	40–46	Ta II (L)	122	129	122	126	120	3.25	120–129
*1b*	92	89	88	87	87	1.85	87–92	Ti II	152	144	149	142	150	3.77	142–152
*2a*	38	41	39	40	42	1.41	38–42	Ge II	113	106	106	107	108	2.61	106–113
*2b*	29	28	28	29	27	0.75	27–29	TFe II	85	78	80	83	84	2.61	78–85
*3a_1_*	23	26	25	26	23	1.36	23–26	BFe II	76	71	67	66	73	3.72	66–76
*3a_2_*	41	46	44	45	41	2.06	41–46	Tr II	49	44	49	44	46	2.24	44–49
*3b*	52	49	42	44	47	3.54	42–52	Cx II	73	74	69	63	71	3.90	63–74
L	99	104	97	98	96	2.79	96–104	Ta III (H)	16	18	19	20	21	1.72	16–21
W	92	97	94	93	91	2.06	91–97	Ta III (L)	143	140	137	133	139	3.32	133–143
AW	75	79	73	69	74	3.22	69–79	Ti III	219	216	217	209	211	3.77	209–219
PW	86	90	86	82	87	2.56	82–90	Ge III	129	123	125	120	126	3.01	120–129
MA	32	33	30	28	29	1.85	28–33	TFe III	106	96	102	100	107	4.02	96–107
AA	14	13	12	13	12	0.75	12–14	BFe III	87	83	86	84	86	1.47	83–87
SB	14	14	13	13	15	0.75	13–15	Tr III	54	53	54	53	47	2.64	47–54
ISD	59	54	50	50	54	3.32	50–59	Cx III	82	77	70	76	80	4.10	70–82
AP	14	14	12	14	16	1.26	12–16	Leg I	699	689	689	698	696	4.35	689–699
AL	71	74	70	69	71	1.67	69–74	Leg II	670	646	642	631	652	12.87	631–670
PL	76	79	75	80	78	1.85	75–80	Leg III	820	788	791	775	796	14.74	775–820
ASE	38	39	36	38	33	2.14	33–39	IP	2189	2123	2122	2104	2144	29.19	2104–2189
PSE	54	50	49	46	47	2.79	46–54	σ	36	37	33	32	35	1.85	32–37
PaScFed	66	73	68	69	74	3.03	66–74	φ’I	22	27	26	23	26	1.94	22–27
PaScGed	50	47	47	54	51	2.64	47–54	φ’’I	51	48	53	47	52	2.32	47–53
*as*	4	4	5	4	4	0.40	4–5	φ’–φ’’I	36	31	34	28	31	2.76	28–36
*bs*	44	47	41	40	43	2.45	40–47	ωI	97	99	92	96	94	2.42	92–99
*cs*	29	30	27	30	29	1.10	27–30	φ’II	17	16	16	19	16	1.17	16–19
GL	177	173	176	175	176	1.36	173–177	φ’’II	30	35	29	31	35	2.53	29–35
Ta I (H)	29	24	24	23	26	2.14	23–29	φ’–φ’’II	124	118	122	120	119	2.15	118–124
Ta I (L)	136	134	132	131	135	1.85	131–136	ωII	22	26	27	23	24	1.85	22–27
								φ’III	28	33	31	32	34	2.06	28–34

**Table 4:** Measurements of *Leptus (Leptus) striatus* **sp. nov.** (larvae, *n* = paratypes).

Character	Holotype	*n* = 17	SD	Range	Character	Holotype	*n* = 17	SD	Range
FD	52	52–56	1.01	52–56	Ti I	191	177–203	7.02	177–203
FV	18	18–20	0.89	18–20	Ge I	132	119–139	4.80	119–139
NDV	70	70–76	1.54	70–76	TFe I	96	94–108	4.32	94–108
IL	457	300–1083	185.74	300–1083	BFe I	110	103–122	5.02	103–122
IW	285	209–764	133.69	209–764	Tr I	59	42–60	5.04	42–60
DS	43–77	39–87	3.14–4.81	39–87	Cx I	63	51–65	3.66	51–65
PDS	47–67	45–77	3.66–4.08	45–77	Ta II (H)	21	17–27	2.22	17–27
Oc	18	15–23	2.09	15–23	Ta II (L)	105	96–116	4.88	96–116
*1a*	87	79–93	4.07	79–93	Ti II	146	139–160	5.68	139–160
*1b*	88	83–99	3.97	83–99	Ge II	105	90–103	4.09	90–103
*2a*	51	45–54	2.48	45–54	TFe II	76	76–86	2.95	76–86
*2b*	49	47–55	2.48	47–55	BFe II	77	77–91	3.83	77–91
*3a_1_*	33	30–38	2.17	30–38	Tr II	55	39–52	3.64	39–55
*3a_2_*	50	47–60	3.59	47–60	Cx II	69	62–85	5.65	62–85
*3b*	51	47–58	3.01	47–58	Ta III (H)	19	16–23	1.93	16–23
L	93	86–98	3.13	86–98	Ta III (L)	122	116–130	4.03	116–130
W	87	76–90	3.65	76–90	Ti III	195	186–219	8.76	186–219
AW	67	62–71	2.45	62–71	Ge III	106	102–123	5.29	102–123
PW	79	70–83	3.32	70–83	TFe III	91	87–102	3.73	87–102
MA	30	26–33	1.95	26–33	BFe III	94	92–109	4.07	92–109
AA	12	10–15	1.16	10–15	Tr III	42	41–53	3.27	41–53
SB	11	11–15	1.13	11–15	Cx III	72	64–80	4.41	64–80
ISD	48	47–58	3.07	47–58	Leg I	775	728–810	22.45	728–810
AP	13	11–15	1.21	11–15	Leg II	633	600–683	16.52	600–683
AL	81	72–88	3.83	72–88	Leg III	722	710–800	23.01	710–800
PL	84	77–89	3.25	77–89	IP	2130	2038–2291	57.59	2038–2291
ASE	70	66–75	2.68	66–75	σ	20	17–31	3.64	17–31
PSE	83	79–92	3.23	79–92	φ’I	14	15–24	2.81	14–24
PaScFed	79	68–82	3.93	68–82	φ’’I	23	23–32	2.70	23–32
PaScGed	53	47–59	2.81	47–59	φ’–φ’’I	27	23–29	1.81	23–31
*as*	3	3–5	0.60	3–5	ωI	22	19–27	1.98	19–27
*bs*	38	31–42	2.79	31–42	φ’II	10	10–12	0.60	10–12
*cs*	17	16–25	2.36	16–25	φ’’II	20	19–29	2.45	19–29
GL	159	153–177	6.00	153–177	φ’–φ’’II	120	114–132	5.52	114–132
Ta I (H)	22	19–25	1.77	19–25	ωII	18	19–23	1.37	18–23
Ta I (L)	124	123–139	5.17	123–139	φ’III	22	21–31	2.93	21–31

## Data Availability

All data are available in this paper.
