# Correction: Xu et al. Four New Species of Larval *Charletonia* and *Leptus* (Acari: Trombidiformes: Erythraeidae), with a Checklist of the Two Genera and Their Hosts from China. *Insects* 2022, *13*, 1154

**DOI:** 10.3390/insects16090912

**Published:** 2025-09-01

**Authors:** Si-Yuan Xu, Tian-Ci Yi, Jian-Jun Guo, Dao-Chao Jin

**Affiliations:** 1 Institute of Entomology, Guizhou University, Guiyang 550025, China; 2 The Guizhou Provincial Key Laboratory for Plant Pest Management of Mountainous Region, Guiyang 550025, China; 3 The Scientific Observing and Experimental Station of Crop Pest in Guiyang, Ministry of Agriculture P. R. China, Guiyang 550025, China

## Text Correction

In the original publication [1], the ZooBank unique identification code (LSID: Life Science Identifier) is missing. To assist readers in locating the newly added ZooBank unique identifiers, we have placed them in the front note Section.

The LSID of the article:

urn:lsid:zoobank.org:pub:528BE312-B0BB-420B-95A8-25B22CE0C540

The LSID of the *Charletonia rectangia* **sp. nov.**:

urn:lsid:zoobank.org:act:C393F8C8-4391-41E2-93DD-A396C2DF65C7

The LSID of the *Leptus* (*Leptus*) *bomiensis* **sp. nov.**:

urn:lsid:zoobank.org:act:24703EFB-5E98-4FEB-9A0A-0EE405E3363C

The LSID of the *Leptus* (*Leptus*) *longisolenidionus* **sp. nov.**:

urn:lsid:zoobank.org:act:2BFDF390-67FF-4C88-9E61-2F90B9990FB7

The LSID of the *Leptus* (*Leptus*) *striatus* **sp. nov.**:

urn:lsid:zoobank.org:act:8867DE72-2DBF-4FE5-9A76-11A31D55907C

The authors state that the scientific conclusions are unaffected. This correction was approved by the Academic Editor. The original publication has also been updated.
